# Osteoporosis and coronary heart disease: a bi-directional Mendelian randomization study

**DOI:** 10.3389/fendo.2024.1362428

**Published:** 2024-05-22

**Authors:** Junsheng Zhang, Pai Xu, Rongcan Liu, Jin Min Gyu, Peng Cao, Chan Kang

**Affiliations:** ^1^ Department of Orthopedic Surgery, Chungnam National University School of Medicine, Daejeon, Republic of Korea; ^2^ Burn & Trauma Treatment Center, Affiliated Hospital of Jiangnan University, Wuxi, China

**Keywords:** osteoporosis, cardiovascular disease, bidirectional, two-sample Mendelian randomization study, causality

## Abstract

**Background:**

Osteoporosis (OP) and cardiovascular disease (CVD) are major global public health issues, especially exacerbated by the challenges of an aging population. As these problems intensify, the associated burden on global health is expected to increase significantly. Despite extensive epidemiological investigations into the potential association between OP and CVD, establishing a clear causal relationship remains elusive.

**Methods:**

Instrumental variables were selected from summary statistics of the IEU GWAS database. Five different components of BMD (heel BMD, LS BMD, FA BMD, FN BMD, and TB BMD) were used as OP phenotypes. CHD, MI, and stroke were selected to represent CVD. Multiple analysis methods were used to evaluate the causal relationship between CVD and OP comprehensively. In addition, sensitivity analyses(Cochran’s Q test, MR-Egger intercept test, and “leave one out” analysis) were performed to verify the reliability of the results.

**Results:**

The MR showed a significant causal relationship between CHD on heel BMD and TB BMD; in the reverse analysis, there was no evidence that OP has a significant causal effect on CVD. The reliability of the results was confirmed through sensitivity analysis.

**Conclusion:**

The study results revealed that CHD was causally associated with Heel BMD and TB BMD, while in the reverse MR analysis, the causal relationship between OP and CVD was not supported. This result posits CHD as a potential etiological factor for OP and prompts that routine bone density assessment at traditional sites (forearm, femoral neck, lumbar spine) using DAX may inadequately discern underlying osteoporosis issues in CHD patients. The recommendation is to synergistically incorporate heel ultrasound or DAX for total body bone density examinations, ensuring clinical diagnostics are both precise and reliable. Moreover, these findings provide valuable insights for public health, contributing to the development of pertinent prevention and treatment strategies.

## 1 Introduction

Cardiovascular disease (CVD) stands as a predominant contributor to global morbidity and mortality. The prevalence of CVD nearly doubled from 1990 to 2019, rising from 271 million to 523 million. Concurrently, deaths from CVD surged from 12.1 million to 18.6 million (1). Notably, in 2019, CVD emerged as the primary cause of death in Asia, claiming 10.8 million lives and representing approximately 35% of total fatalities in the region (2). According to the most recent published reports by the American College of Cardiology, the global incidence of cardiovascular diseases witnessed a 29.01% increase over the past decade, culminating in 607.64 million cases in 2020. Correspondingly, the death toll rose to 19.05 million, marking an 18.71% surge over the same period (3). While the current trajectory of CVD prevention appears promising, there remains a compelling imperative to formulate and implement effective monitoring and prevention strategies aimed at alleviating the burden of CVD, particularly in underserved global populations (4). Additionally, with a trend toward younger onset of CVD, a pressing need exists to comprehensively comprehend the pathogenesis of CVD to confront this inevitable challenge (5).

Osteoporosis (OP), a metabolic bone disorder stemming from a convergence of multifactorial elements, may result in a reduction of bone density in mild cases, while in severe instances, it can culminate in fractures. The fundamental etiology of the condition lies in the dysregulation between bone formation and resorption processes (6, 7). Epidemiological data underscores a staggering global prevalence of osteoporosis is 19.7%, and osteopenia is as high as 40.4% (8). Projections based solely on population aging portend a substantial escalation in the majority of osteoporosis and fragility fractures in the coming decade (9). Regrettably, a worldwide survey targeting older demographics reveals an osteoporosis prevalence of 21.7%, with the highest rates observed in Asian countries at 24.3%, succeeded by Europe (16.7%) and the United States (11.5%) (10). In light of these findings, some scholars boldly advocate for universal osteoporosis assessment and intervention, positing it as a requisite measure to mitigate the direct and indirect global burden imposed by osteoporosis (11). Evidently, osteoporosis’s disconcerting status necessitates urgently exploring its causative factors to curtail its global impact.

OP and CVD commonly coexist in clinical settings. Despite numerous observational studies attempting to ascertain the precise relationship between them, a definitive consensus remains elusive. Given this context, the imperative to clarify the causal link between OP and CVD becomes even more pronounced. Not only does this hold clinical significance, but it also fortifies our preparedness to confront ensuing challenges. Observational studies, susceptible to confounding factors, need to ascertain the causal relationship between OP and CVD more adequately. To circumvent these limitations, Mendelian randomization (MR) emerges as a potent method for causal inference, utilizing genetic variation as an instrumental variable (IV). This approach effectively mitigates confounding biases inherent in traditional epidemiological research (12). Consequently, to establish the causal relationship between OP and CVD definitively, we conducted a bi-directional MR study.

## 2 Materials and methods

### 2.1 Research design

**Figure 1:**
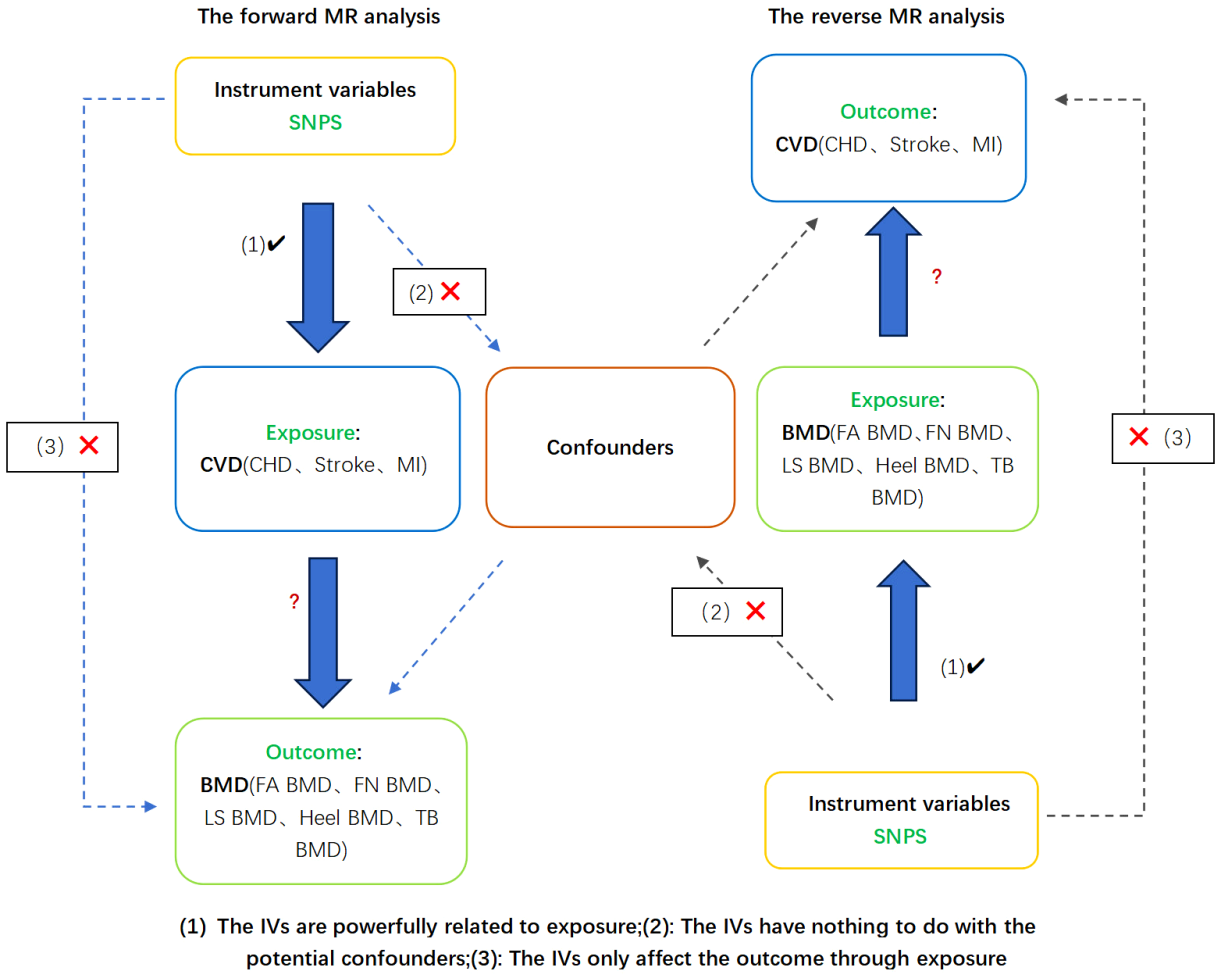
Design and hypotheses of a bi-directional Mendelian randomization study of causality in cardiovascular disease and osteoporosis. MR, Mendelian randomization. CHD, coronary heart disease; MI, myocardial infarction; FA BMD, Forearm bone mineral density; FN BMD, Femoral neck bone mineral density; LS BMD, Lumbar spine bone mineral density; TB BMD, Total body bone mineral density.

This study adheres to the methodological tenets outlined in the Guideline (13). Employing bi-directional Mendelian randomization (MR) analysis involving two datasets, we sought to delineate the directional causality between CVD and OP, encompassing diverse analytical facets. Initially, we scrutinized potential causal associations between CVD (coronary heart disease [CHD], myocardial infarction [MI], Stroke) and OP indicators (total body BMD [TB BMD], lumbar spine BMD [LS BMD], forearm BMD [FA BMD], femoral neck BMD [FN BMD], Heel BMD). Subsequently, we conducted a reciprocal analysis in the opposite direction. The bi-directional MR analysis involving two datasets is depicted in **Figure 1** below. All data used in this study were obtained from free and open databases or existing publications and did not require ethical approval to be conducted.

### 2.2 Data sources

OP and cardiovascular disease CVD exhibit a substantial hereditary component, with evidence suggesting heritability rates of up to 40% to 60% for coronary artery disease and 60% to 80% for BMD (14, 15). This hereditary influence poses significant challenges to our endeavors to alleviate the burdens imposed by these conditions. The most recent Global Burden of Disease (GBD) statistics underscore the pivotal role of ischemic heart disease (IHD) and stroke in contributing to the overall burden of CVD (1). IHD, predominantly manifesting as coronary artery disease, serves as the primary pathological process underlying IHD, with the terms often employed interchangeably (16). Acute cardiovascular events, including common strokes and myocardial infarctions, constitute emergencies within the spectrum of CVD (5). Consequently, representative CVDs such as CHD, stroke, and MI were selected for investigation. Osteoporosis diagnosis, as per the World Health Organization (WHO), is established when BMD measured at the spine, hip, or wrist falls more than 2.5 standard deviations below the average BMD reference value for young adults (17). Currently, dual-energy X-ray absorptiometry (DXA) serves as the standard clinical method for detecting osteoporosis. Despite its widespread use, DXA measurements may yield errors for individuals with immature bones, necessitating the adoption of whole-body measurements, particularly in pediatric populations (18). Given the limitations of DXA for large-scale osteoporosis screening, quantitative ultrasound (QUS) emerges as a viable alternative characterized by simplicity, portability, cost-effectiveness, and the absence of ionizing radiation; it is suitable for bone health assessments in diverse populations, including young children. Furthermore, when juxtaposed with DXA for fracture prediction, the overall advantages of QUS are relatively apparent (19). Additionally, heel bone density estimation using ultrasound has high heritability and a strong correlation with DXA-based bone density (20). Consequently, our selection of LS BMD, FA BMD, and FN BMD measured by DXA, along with TB BMD and Heel BMD, as representative components for OP aimed to guarantee the reliability and persuasiveness of the chosen BMD parameters. In assembling the largest GWAS database to date, encompassing three separate GWAS summary statistics for LS BMD (n = 28,498), FN BMD (n = 32,735), and FA BMD (n = 8143), our study stands at the forefront of DXA-measured BMD research. While age is a recognized common risk factor for both OP and CVD, the majority of prior investigations have concentrated on adults, particularly older women. However, compelling evidence indicates that children with congenital heart disease are also susceptible to severe metabolic bone disease and fragility-related fractures (21). Motivated by this insight, our study explored the potential age-specific relationship between CVD and TB BMD across five distinct age groups (0–15, 15–30, 30–45, 45–60, and over 60 years old) as detected by DXA. This targeted approach aims to facilitate early and precise intervention in corresponding age cohorts While fortifying the reliability of our research outcomes. To the best of our knowledge, this study represents the first relatively comprehensive evaluation of Mendelian randomization between CVD and OP. Detailed information on the data employed is available in (**Table 1**).

### 2.3 Genetic instrumental variable selection criteria

To identify Proper single nucleotide polymorphisms(SNPs) of CVD and BMD, our approach in the OPEN GWAS databases involved a meticulous series of steps. Firstly, SNPs demonstrating robust association (*p* < 5E-8), independent inheritance (r2 < 0.001, kb = 10,000), and lack of linkage disequilibrium (LD) were meticulously selected from the GWAS data of CVD or BMD. This curation process was executed through the clump data function within the Two-Sample Mendelian Randomization (MR) package in R software (version 4.3.1). Subsequently, outcome-associated SNPs were systematically eliminated by querying each one individually using the PhenoScanner database (http://www.phenoscanner.medschl.cam.ac.uk). Following this, SNPs featuring palindromic alleles and incompatible variations were systematically excluded to preclude chain ambiguity issues. The remaining SNPs underwent F-statistics calculation to assess the correlation between exposure and SNPs, with the value less than 10 indicating weakness and necessitating elimination (22). The resultant SNPs, which were deemed robust, were employed for the subsequent MR analysis. To evaluate the efficacy of instrumental variables (IVs), F statistics for each SNP were computed using the formula F = R2(N-2)/(1-R2). The calculation formula for R2 is articulated as R2 = (2 x EAF x (1 - EAF) x beta^2)/[(2 x EAF x (1 - EAF) x beta^2) + (2 x EAF x (1 - EAF) x N x (SE(beta)^2))], where EAF represents the effect allele frequency, N is the sample size, beta signifies the estimated impact of the genetic effect on the outcome, and SE denotes the standard error of the genetic effect (23).

### 2.4 MR analysis

To fortify the robustness of our study findings, we employed a comprehensive approach encompassing four distinct methods within the MR analysis framework. These methodologies included inverse variance weighting (IVW), MR-Egger regression, weighted mode, and weighted median. The selection of IVW as the primary analytical method stems from its superior statistical power in scenarios where instrumental variables (IVs) exhibit no pleiotropic effects (24). The utilization of either fixed effects IVW or random effects IVW models depends on the presence or absence of heterogeneity within the dataset (25). In cases over 50% of data is obtained from null instrumental variables, the weighted median approach provides an impartial causal estimate (26). The MR-Egger method, capable of accommodating horizontal pleiotropic effects in all SNPs, serves as a valuable tool for estimating the causal possibility of exposure on the outcome (27). The Bonferroni method was applied to mitigate the risk of false positives resulting from multiple comparisons. Consequently, associations with a P value <0.003 (0.05 divided by 3*5) were deemed significant evidence of a causal link, while the P value less than 0.05 but greater than 0.003 were considered suggestive evidence.

### 2.5 Sensitivity analysis

In order to fortify the reliability of the outcomes derived from MR analysis, a thorough assessment of both heterogeneity and horizontal pleiotropy was meticulously conducted (28). Cochran’s Q test estimates, derived from IVW estimates, were used to rule out heterogeneity among IVs (29). A P value < 0.05 denoted the presence of substantial heterogeneity. The assessment of horizontal pleiotropy and the correction of potential outliers were performed using MR Pleiotropy Residual Sum and Outliers (MR-Presso) (30). A P value < 0.05 in this context was considered indicative of significant horizontal pleiotropy. Additionally, the leave-one-out sensitivity analysis was employed to discern the potential influence of individual SNPs (31).

## 3 Results

### 3.1 Effects of CVD characteristics on BMD in different parts or at different ages

#### 3.1.1 Set the following conditions

The Instrumental Variable Selection (IVS) exhibited no linkage disequilibrium (LD) (r2 < 0.001), adhered to the physical distance threshold (10,000 kb), and possessed genome-wide dominance (*p*< 5E-8). The data extracted from the Genome-Wide Association Study (GWAS) database underwent meticulous screening for Coronary Heart Disease (CHD) (15 SNPs), Myocardial Infarction (MI) (80 SNPs), and Stroke (17 SNPs) within IVS, as outlined in **Supplementary Table 2**; **Supplementary Figure S1**. Subsequently, SNPs associated with Bone Mineral Density (BMD) risk factors were systematically eliminated one by one, aided by the website (http://www.phenoscanner.medschl.cam.ac.uk), with detailed information presented in **Supplementary Table 2**; **Supplementary Figure S2**. Following this, the Minor Allele Frequency (MAF) threshold (>0.01) was applied via the two-sample Mendelian Randomization (MR) function of the R package to exclude palindromic or incompatible SNPs, as elucidated in **Supplementary Table 2**; **Supplementary Figure S3**. F statistics computed for the ultimately obtained SNPs revealed values exceeding 10, affirming the absence of weak instruments. Comprehensive details are available in (**Supplementary Table 2**; **Supplementary Figure S1**).

#### 3.1.2 The causal impact of CVD on BMD in different parts

**Figure 2:**
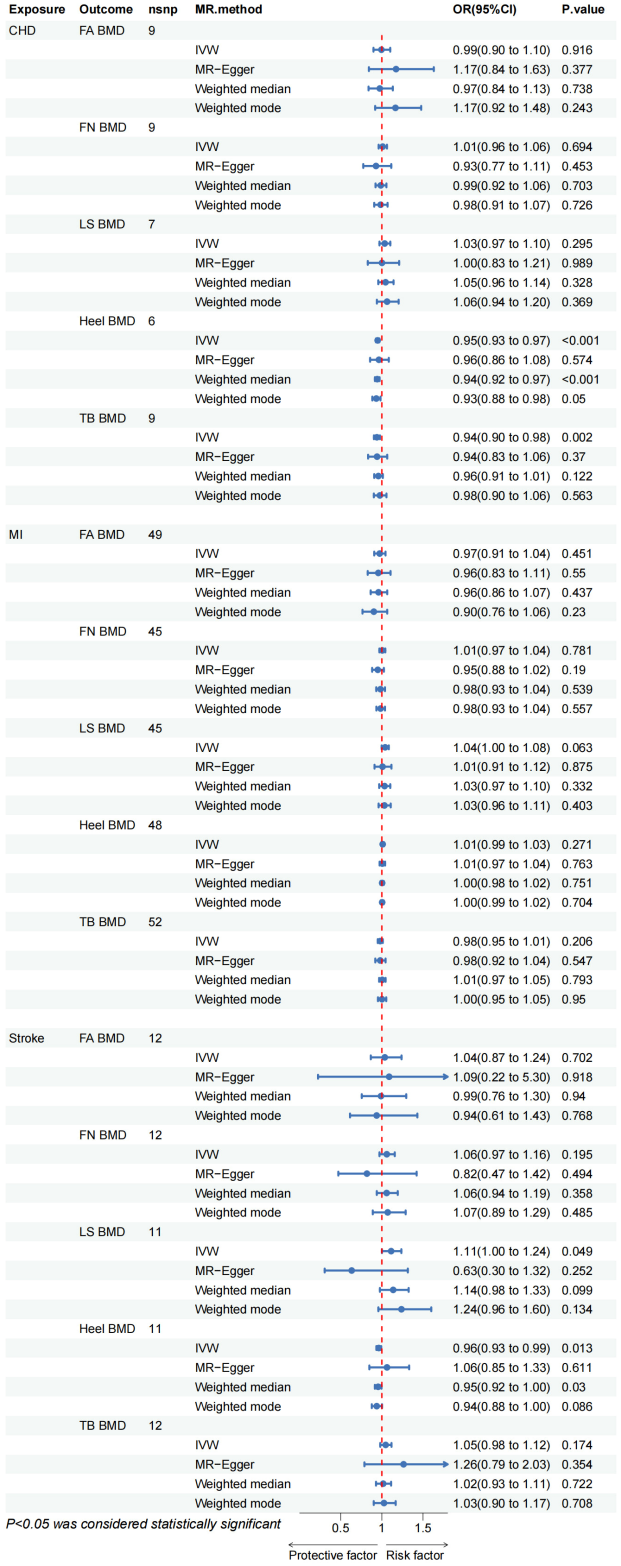
CHD, coronary heart disease; MI, myocardial infarction; FA BMD, Forearm bone mineral density; FN BMD, Femoral neck bone mineral density; LS BMD, Lumbar spine bone mineral density; TB BMD, Total body bone mineral density; IVW, inverse variance weighted.

The outcomes of the IVW method analysis are depicted in **Figure 2**. During the MR-Presso global test evaluating CVD impact on BMD across diverse regions, outliers were identified between CHD and BMD (LS BMD, Heel BMD), between MI and Heel BMD, and between stroke and BMD (LS BMD, Heel BMD) (**Supplementary Table 2**; **Supplementary Figure S4**). Following their removal and subsequent reanalysis, no outliers were observed (**Table 1**). Notably, a substantial causal association emerged between genetically predicted CHD and Heel BMD, as well as Total Body BMD (Heel BMD: Odds Ratio [OR]: 0.949, 95% Confidence Interval [CI]: 0.928–0.970, *p*< 0.001; TB BMD: OR: 0.940, 95% CI: 0.903–0.976, *p*= 0.002), as depicted in **Figure 2**. The beta values direction acquired through IVW analysis aligned consistently with those analyses with the MR-Egger regression, weighted mode, and weighted median. Cochran’s Q test revealed no evidence of heterogeneity in the effects of CHD on Heel BMD and TB BMD (*p* > 0.05) (**Table 2**). Furthermore, MR-Egger intercept tests indicated no horizontal pleiotropy (**Table 2**). Leave-one-out analysis results demonstrated that the IVW outcomes were not forced by potential single SNPs (**Supplementary Table 1**; **Supplementary Figure S8**, **S10**), affirming the significant causal relationship. Similarly, the IVW results indicated suggestive causal associations between Stroke and LS BMD and Heel BMD (LS BMD: OR = 1.113, 95% CI: 1.000–1.237, *p* = 0.049; Heel BMD: OR = 0.962, 95% CI: 0.933–0.992, *p* = 0.013), with related Cochran’s Q test and MR-Egger intercept test P values exceeding 0.05 (**Table 2**). MR-Presso global tests identified no outliers, signifying the absence of heterogeneity and horizontal pleiotropy. However, inconsistencies were noted in the direction of IVW and MR-Egger results for Stroke on Lumbar Spine BMD and Heel BMD (**Supplementary Table 2**; **Supplementary Figure S5**), urging caution regarding the robustness of the suggestive causal relationship.

**Table 1:** The GWAS datasets used for MR analysis.

	Traits	Year	Sample	Population	PMID/Dataset
BMD	FA BMD	2015	8,143	European	26367794
	FN BMD	2015	32,735	European	26367794
	LS BMD	2015	28,498	European	26367794
	Heel BMD	2018	265627	European	ukb-b-8875
	TB BMD	2018	56284	European	29304378
	TB BMD (age 0–15)	2018	11807	Mixed	29304378
	TB BMD (age 15–30)	2018	4180	Mixed	29304378
	TB BMD (age 30–45)	2018	10062	Mixed	29304378
	TB BMD (age 45–60)	2018	18805	European	29304378
	TB BMD (age over 60)	2018	22504	Mixed	29304378
CVD	CHD	2011	86995	European	21378990
	Stroke	2018	446696	Mixed	29531354
	MI	2021	395,795	European	33532862

CHD, coronary heart disease; MI, myocardial infarction; FA BMD, Forearm bone mineral density; FN BMD, Femoral neck bone mineral density; LS BMD, Lumbar spine bone mineral density; TB BMD, Total body bone density.

**Table 2:** Heterogeneity, pleiotropy test and MR-PRESSO Global test of exposure (Cardiovascular diseases) on bone density in different parts of the body.

Exposure	Outcome	Cochran Q statistic	Heterogeneity P-value	MR-Egger Intercept	Intercept p-value	MR-PRESSO Global test P-value
CHD	FA BMD	9.981257188	0.266343882	-0.021708211	0.333103117	0.239
CHD	FN BMD	12.08571868	0.14742011	0.011001709	0.368521369	0.181
CHD	LS BMD	6.002037674	0.422961858	0.004317761	0.731418586	0.438
CHD	Heel BMD	6.223863792	0.285041471	-0.001767004	0.79939395	0.342
CHD	TB BMD	7.502252345	0.483534629	-0.000336768	0.965948036	0.528
MI	FA BMD	39.44951252	0.805513645	0.001558789	0.789557916	0.769
MI	FN BMD	40.67336071	0.615000025	0.00496603	0.102940633	0.642
MI	LS BMD	58.76266838	0.067447088	0.00275713	0.497732763	0.076
MI	Heel BMD	109.195188	7.37644E-07	0.000432389	0.757968854	NA
MI	TB BMD	78.76635325	0.007556748	-4.81662E-05	0.98528366	NA
Stroke	FA BMD	19.49416314	0.052779715	-0.002917025	0.046735383	0.053
Stroke	FN BMD	9.855103431	0.543465987	0.015080821	0.375462836	0.557
Stroke	LS BMD	13.84694923	0.180087894	0.032675732	0.160174796	0.188
Stroke	Heel BMD	14.43156378	0.154200894	-0.005762571	0.405716145	0.186
Stroke	TB BMD	13.81145052	0.243602861	-0.011021547	0.445767771	0.264

CHD, coronary heart disease; MI, myocardial infarction; FA BMD, Forearm bone mineral density; FN BMD, Femoral neck bone mineral density; LS BMD, Lumbar spine bone mineral density; TB BMD, Total body bone mineral density.

The IVW method proves that no discernible causal relationship was identified between other CVDs and BMD at different sites (*p* > 0.05). Due to heterogeneity between MI and TB BMD and Heel BMD (*p* < 0.05) (**Table 2**), the random-effects IVW method was employed for analysis. Rigorous sensitivity analyses confirmed the steadfastness of these Mendelian Randomization effect estimates (**Table 2**). Detailed scatter plots, leave-one-out analyses, forest plots, and funnel plots elucidating the causal relationship between the remaining CVD and BMD at various sites are available in **Supplementary Table 1**; **Supplementary Figures S1**-**S20**.

#### 3.1.3 The causal impact of CVD on BMD at different ages

**Figure 3:**
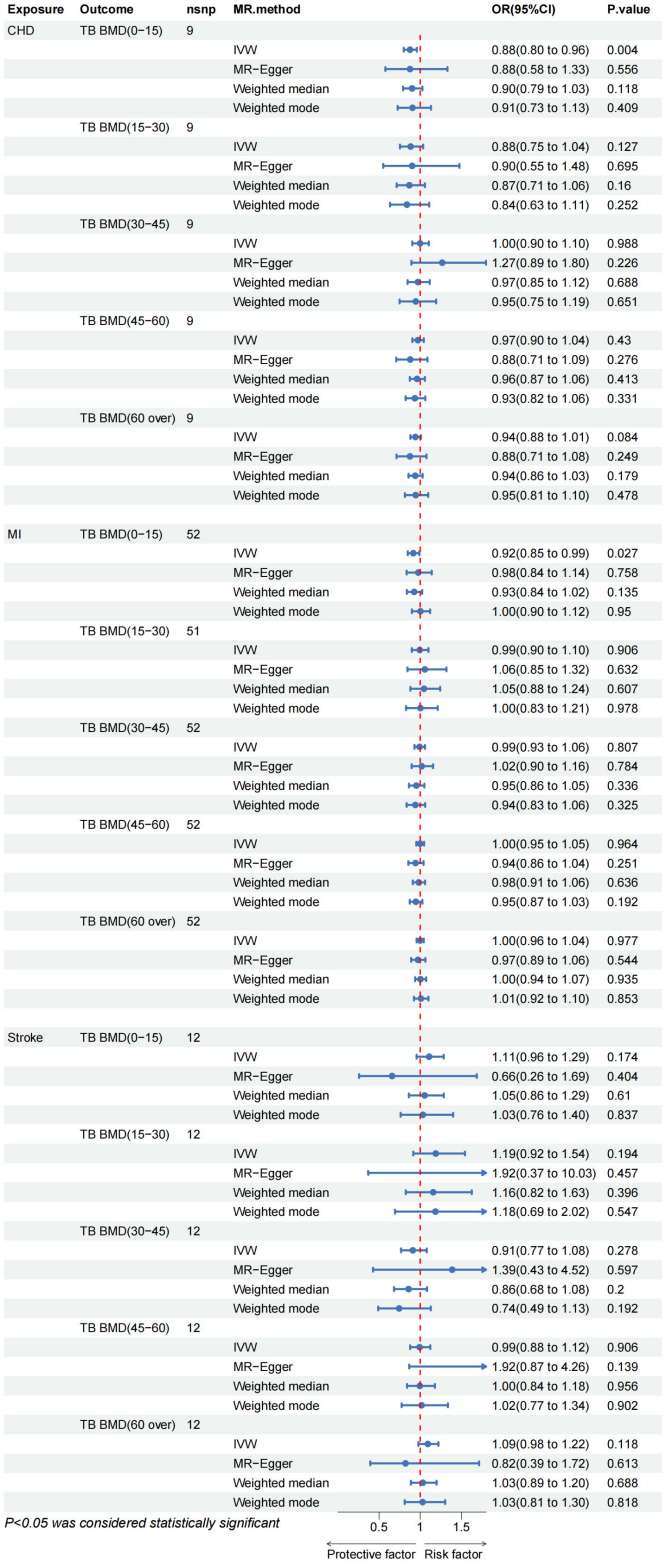
CHD, coronary heart disease; MI, myocardial infarction; FA BMD, Forearm bone mineral density; FN BMD, Femoral neck bone mineral density; LS BMD, Lumbar spine bone mineral density; TB BMD, Total body bone mineral density; IVW, inverse variance weighted.

The results of the IVW test suggest a potential causal association between CHD and TB BMD in the age group of 0–15 years (OR: 0.876, 95% CI: 0.800–0.959, p= 0.004> 0.003) (**Figure 3**). Cochran’s Q test revealed no marked heterogeneity (*p* > 0.05) (**Table 3**). Both MR-Egger regression and the assessment of horizontal pleiotropy through the MR-Presso global test for CHD and TB BMD (0–15 years old) indicated no compelling evidence of horizontal pleiotropic effects (**Table 3**). Leave-one-out test confirmed the result’s robustness against the influence of any single SNP driver (**Supplementary Table 1**; **Supplementary Figure S26**). Detailed scatter plots, forest maps, and funnel plots illustrating the relationship between CHD and TB BMB(0-15 years old) are provided in **Supplementary Table 1**; **Supplementary Figure S21, S31, S36**. Similarly, it can be seen that there is a potential causal relationship between MI and TB BMD in the age group of 0-15 years (OR: 0.917, 95% CI: 0.849-0.990, p= 0.027> 0.003) (**Figure 3**).

**Table 3:** Heterogeneity, pleiotropy test and MR-PRESSO Global test of exposure (Cardiovascular diseases) on bone density in different ages of the body.

Exposure	Outcome	Cochran Q statistic	Heterogeneity P-value	MR-Egger Intercept	Intercept p-value	MR-PRESSO Global test P-value
CHD	TB BMD(0–15)	15.24543109	0.054545261	-3.68793E-05	0.998895535	0.069
CHD	TB BMD(15–30)	1.62705036	0.99039186	-0.002622299	0.933568547	0.989
CHD	TB BMD(30–45)	11.50377187	0.17475509	-0.030391399	0.201769059	0.189
CHD	TB BMD(45–60)	5.791901082	0.670529056	0.01298506	0.362509046	0.666
CHD	TB BMD(over 60)	6.310036709	0.612546448	0.009398325	0.486422765	0.607
MI	TB BMD(0–15)	91.04625989	0.000481157	-0.005731015	0.370706372	NA
MI	TB BMD(15–30)	61.42786061	0.128963805	-0.005689807	0.536746683	0.146
MI	TB BMD(30–45)	46.17481527	0.66535308	-0.002433807	0.646606169	0.644
MI	TB BMD(45–60)	55.30637531	0.315410365	0.00525933	0.194744373	0.354
MI	TB BMD(over 60)	46.29174082	0.660843888	0.002462153	0.496651743	0.63
Stroke	TB BMD(0–15)	5.662353483	0.894916813	0.030383226	0.297748447	0.901
Stroke	TB BMD(15–30)	6.960129909	0.802303007	-0.028194446	0.577522332	0.794
Stroke	TB BMD(30–45)	12.46657314	0.329612743	-0.024649746	0.493198086	0.338
Stroke	TB BMD(45–60)	13.1239661	0.285300354	-0.03850296	0.130545905	0.288
Stroke	TB BMD(over 60)	11.49791518	0.40253988	0.016636366	0.459810141	0.408

CHD, coronary heart disease; MI, myocardial infarction; FA BMD, Forearm bone mineral density; FN BMD, Femoral neck bone mineral density; LS BMD, Lumbar spine bone mineral density; TB BMD, Total body bone density.

Other IVW results indicated no evidence of a significant causal relationship between various CVD and TB BMD across different age groups (**Figure 3**). While outliers were initially identified in the MR-Presso global test for MI on TB BMD in the age group of 15–30 years, subsequent removal of outliers (**Supplementary Table 2**; **Supplementary Figure S4**) did not alter the findings. Sensitivity analysis further supported the stability of these results (**Table 3**). Additional visualization results are presented in **Supplementary Table 1**. In summary, there is no discernible proof supporting a causal rapport between CVD and TB BMD across different age groups.

### 3.2 Impact of OP characteristics on CVD

#### 3.2.1 Set the following conditions

The Instrumental Variable Selection (IVS) exhibits no linkage disequilibrium (r2 < 0.001), adheres to the physical distance threshold (10,000 kb), and possesses significant genome-wide prominence (*p* < 5E-8). Our comprehensive screening of IVS for TB BMD (85 SNPs), FA BMD (3 SNPs), FN BMD (21 SNPs), LS BMD (24 SNPs), and Heel BMD (359 SNPs) is outlined in **Supplementary Table 2**; **Supplementary Figure S1**. Subsequently, aided by professional tools (http://www.phenoscanner.medschl.cam.ac.uk), we manually removed SNPs connection with risk factors for different BMDs, detailed in **Supplementary Table 2**; **Supplementary Figure S2**. The Minor Allele Frequency (MAF) threshold (> 0.01) was applied using the two-sample Mendelian Randomization (MR) function settings of the R package to eliminate outliers (**Supplementary Table 2**; **Supplementary Figure S3**). F statistics calculated for the ultimately obtained SNPs revealed values all exceeding 10, affirming the absence of weak instruments. Detailed information is provided in **Supplementary Table 2**; **Supplementary Figure S1**.

#### 3.2.2 Causal impact of OP on CVD

**Figure 4:**
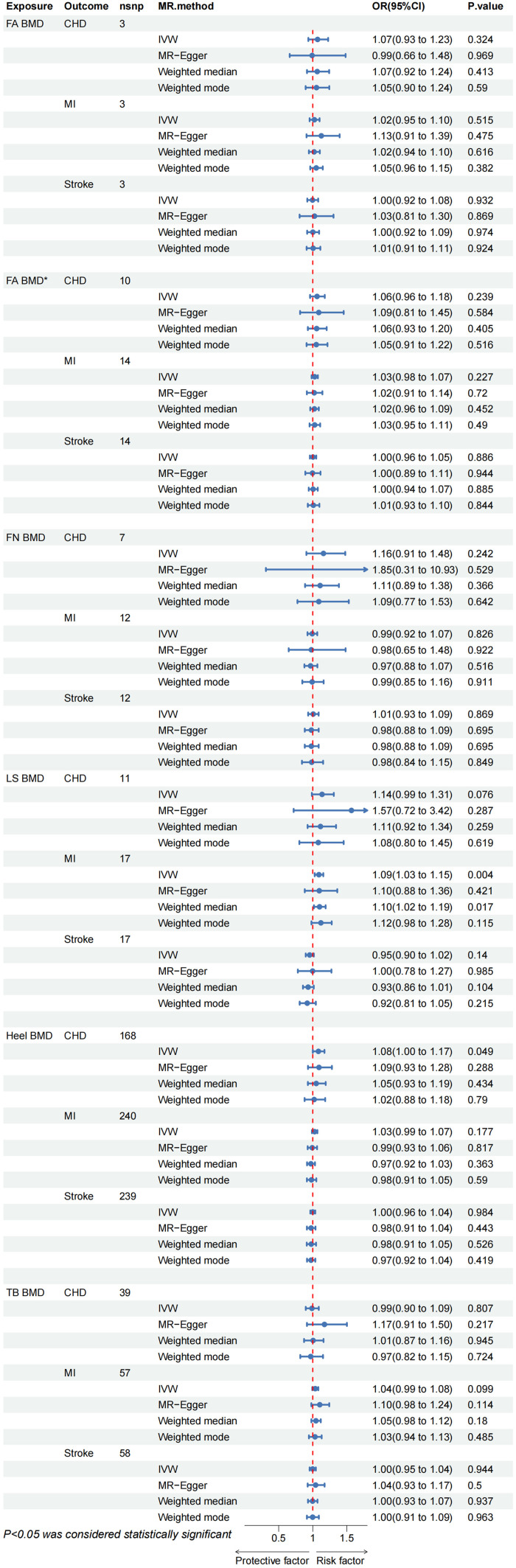
CHD, coronary heart disease; MI, myocardial infarction; FA BMD, Forearm bone mineral density(p < 5E-8); FA BMD*, Forearm bone mineral density(p < 5E-6); FN BMD, Femoral neck bone mineral density; LS BMD, Lumbar spine bone mineral density; TB BMD, Total body bone mineral density; IVW, inverse variance weighted.

The Reverse Mendelian Randomization (MR) analysis is depicted in **Figure 4**. Overall, no evidence of a reverse causal relationship between OP indicators (Heel BMD, TB BMD, LS BMD, FA BMD, FN BMD) and CVD (CHD, MI, Stroke) was observed. Only a suggestive causal association emerged between Heel BMD and CHD (OR= 1.083, 95% CI: 1.000–1.172, *p* = 0.049), as well as LS BMD and MI (OR= 1.090, 95% CI: 1.029–1.155, *p* = 0.004). In the sensitivity analysis, only an abnormal SNP association between Heel BMD and TB BMD on MI was identified (**Supplementary Table 2**; **Supplementary Figure S4**). Upon removal and re-analysis, no evidence of horizontal pleiotropy persisted (**Table 4**). Cochran’s Q test discovered heterogeneity in the causal impact of Femoral Neck BMD on CHD (*p* = 0.041) and Heel BMD on MI (*p*< 0.001). To address this heterogeneity, a random-effects IVW analysis was employed, ensuring the robustness and reliability of the IVW results. Additionally, leave-one-out analysis yielded no evidence of potential SNP-driven influences (**Supplementary Table 1**; **Supplementary Figures S44**-**S46**). Detailed scatter plots, forest maps, and funnel plots related to these findings can be found in **Supplementary Table 1**.

**Table 4:** Heterogeneity, pleiotropy test, and MR-PRESSO Global test of exposure (Bone density) on cardiovascular diseases.

Exposure	Outcome	Cochran Q statistic	Heterogeneity P-value	MR-Egger Intercept	Intercept p-value	MR-PRESSO Global test P-value
FA BMD	CHD	0.17724079	0.915192919	0.010774879	0.755795167	
FA BMD	MI	0.842662038	0.65617286	-0.012808958	0.52730918	
FA BMD	Stroke	0.3326947	0.846752064	-0.003861565	0.843207372	
FA BMD*	CHD	5.469318279	0.791629648	-0.002911733	0.869737391	0.843
FA BMD*	MI	9.538775337	0.731144943	0.000877126	0.89910032	0.755
FA BMD*	Stroke	11.60054438	0.560639223	0.001009422	0.887301414	0.535
FN BMD	CHD	13.13664354	0.040916632	-0.032766747	0.624154903	0.059
FN BMD	MI	11.22098217	0.424940202	0.000872913	0.950565157	0.429
FN BMD	Stroke	12.46940067	0.329412951	-0.013581485	0.375703225	0.358
LS BMD	CHD	11.67929149	0.307093553	-0.022747696	0.430148672	0.357
LS BMD	MI	12.75957677	0.690248768	-0.000394365	0.961316768	0.687
LS BMD	Stroke	11.80310411	0.757418946	-0.003313896	0.717270374	0.765
Heel BMD	CHD	197.3724078	0.054068931	-0.000298103	0.910782468	0.058
Heel BMD	MI	315.2117255	0.000683149	0.0014408	0.234462054	NA
Heel BMD	Stroke	235.0078903	0.542655969	0.001035209	0.361120025	0.513
TB BMD	CHD	41.39537014	0.324748774	-0.009833151	0.149198444	0.332
TB BMD	MI	66.23340869	0.164585572	-0.003632699	0.274125272	0.641
TB BMD	Stroke	53.59576291	0.603573546	-0.002499529	0.448277601	0.61

CHD, coronary heart disease; MI, myocardial infarction; FA BMD*, Forearm bone mineral density (p<5E-6); FN BMD, Femoral neck bone mineral density; LS BMD, Lumbar spine bone mineral density; TB BMD, Total bone mineral density.

Due to insufficient variables for FA BMD, MR-Presso analysis could not be conducted. Nevertheless, the MR multi-effect residual P-value exceeded 0.05, and the identified Instrumental Variables (IVs) have been previously applied in related MR studies (32, 33). Furthermore, screening conditions from the same database with *p*< 5E-6, r2 < 0.001, and kb = 10000 were employed to obtain additional FA BMD IVs (16 SNPs). No evidence of causal association was observed (**Figure 4**), and sensitivity analysis confirmed the stability of the results (**Table 4**). In summary, we assert that there is no compelling evidence supporting a causal relationship between OP and CVD, affirming the reliability of our findings.

## 4 Discussion

In this bidirectional MR study, we assessed the causal association between CVD and OP. The results indicate a causal relationship between genetically predicted CHD and Heel BMD, as well as TB BMD. Reverse MR analysis found no evidence linking genetic predispositions for BMD in different anatomical sites to these CVDs. Although we did not observe a significant causal relationship between the genetic predisposition to BMD at different sites and these CVD diseases, we did observe a significant causal association between CHD with Heel BMD and TB BMD. Initially, we conducted thorough sensitivity analyses to validate adherence to the three fundamental assumptions inherent in MR. The outcomes of the MR study, employing diverse methodologies, consistently exhibited coherence. Subsequently, we systematically examined each instrumental variable independently to alleviate the impact of confounding factors. This meticulous scrutiny involved a step-by-step screening of instrumental variables to minimize the potential interference of confounding factors. Given that FA BMD presented fewer instrumental variables and the possibility of outliers could not be ruled out, we undertook rigorous efforts to substantiate this aspect. Ultimately, we applied the rigorous Bonferroni method to address multiple comparisons, eliminating the risk of false positive results. Consequently, our MR findings stand as robust and reliable, ensuring the validity of the study outcomes.

As prevalent public health issues with widespread impact and significant consequences, OP and CVD have consistently garnered considerable attention. While numerous prior epidemiological studies have reported the connection between these conditions, the precise association remains elusive (34–43). Several factors may contribute to this ambiguity. Firstly, common risk factors such as age, vitamin D deficiency, inactivity, smoking, and diabetes are shared between osteoporosis and CVD (44). Early investigations highlighted a potential “bone-vascular axis,” and subsequent research recognized shared pathogenic processes involving oxidative stress, inflammation, and lipid metabolism, mediated by common regulatory factors like bone morphogenetic protein (BMP), osteopontin (OPN), matrix GLA protein (MGP), proinflammatory cytokines (IL-6 and tumor necrosis factor-α (TNFα)), sclerostin, gamma-carboxy glutamic acid-rich matrix (GLA) protein (MGP), and fibroblast growth factor (FGF)-23 (45–49). Additionally, pathways such as the RANKL/RANK/OPG (osteoprotegerin) and Wnt signaling are implicated in the pathogenesis of vascular calcification and cardiovascular disease (50). Hyperhomocysteinemia has also been identified as a contributor to both vascular and bone diseases (51), emphasizing a shared pathological basis between OP and CVD. Furthermore, observations of patients’ drug use and diet have reinforced the link between the two conditions. For instance, anticoagulants like warfarin and unfractionated heparin, commonly used in stroke and myocardial infarction patients, may increase the risk of OP (52). Conversely, excessive calcium supplements, particularly in a calcium-sufficient diet, have been associated with elevated cardiovascular risk, especially myocardial infarction risk (53). Statins, commonly used lipid-lowering drugs in CVD treatment, have been linked to bone changes (54). Various medications, including sclerostin-targeted drugs, SERMs, hypoglycemic drugs, antihypertensive drugs, selective estrogen receptor modulators, and anti-bone resorption drugs, have demonstrated effects on both OP and CVD. Even specific treatments like vitamin D have shown potential in reducing CVD risk (55, 56). Finally, the reliability of results from prior studies is constrained by divergent methodologies and populations (57). Notably, a substantial portion of past observational inquiries has disproportionately focused on the elderly, particularly postmenopausal women, introducing inherent crowd bias that undermines result generalizability. Moreover, inadequacies in sample sizes, potential bias in article selection, and methodological disparities can induce instability in research outcomes. For the first time, we posit CHD as a potential cause of OP from a genetic perspective. Acknowledging that MR studies may not represent the pinnacle of evidence-based medicine, we anticipate that future investigations with higher evidential levels will corroborate our findings. Nevertheless, in juxtaposition with observational research, the clinical significance of our MR results remains considerable. Simultaneously, in auxiliary examinations, conventional anatomical sites (forearm, femoral neck, lumbar spine) can be utilized for Dual-Energy DAX examination. Vice versa, for instance, Romosozumab, approved by the FDA, is accompanied by a black box warning indicating a potential increase in the risk of cardiovascular disease (58). If an osteoporotic patient requires the use of this medication for treatment, clinicians are encouraged to make a decision regarding the drug based on the individual patient’s specific condition, even if cardiovascular disease is present. Conversely, disapprove that the use of the drug’s potential risks is overly interpreted as an absolute contraindication, instigating apprehension and reluctance towards adoption. It is noteworthy that, following the prevailing gold standards in osteoporosis (OP) diagnosis—utilizing FN BMD, FA BMD, and LS BMD—our results are similar to the MR results of HE B, Gua C, and Bhatta L and others (32, 59). This not only bolsters the robustness of our research findings but also underscores pertinent clinical considerations. Grounded in the established diagnostic approach of DAX presently in common use, it may not sufficiently discern potential skeletal issues in patients with CHD. Particularly within adult cohorts enduring congenital heart disease over an extended duration, susceptibility to bone diseases is heightened (60, 61). Consequently, we advocate that, under the precondition of examining bone density in conventional anatomical regions (forearms, femoral neck, lumbar spine) through DAX for CHD patients, if circumstances allow, simultaneous consideration should be given to bone density assessments via heel ultrasound or DAX throughout the body. This holistic approach is good for preventing and treating skeletal diseases with them. As highlighted earlier, the advantages of bone ultrasound render it a preferred choice. Furthermore, our MR results offer valuable insights for public health in crafting comprehensive prevention and treatment strategies. These strategies stand to exert positive effects on a wide population, ultimately contributing to the effective management of the substantial burden imposed by both osteoporosis and cardiovascular diseases.

Our study has several limitations. First, this study is primarily from a European population, and our findings cannot be generalized to other populations. Second, due to the limited exposure variance explained by the SNP instrument or the limited sample size of the resulting GWAS (for example, there are only 3 SNPs with strong FA BMD correlations we extracted), it may lead to weak instrumental variable bias, so we relax the settings Conditional extraction of more FA BMD SNPs replicated the reliability of the results. However, larger-scale GWAS are still needed to enhance the ability of correlational MR studies to detect associations. Third, due to the limitations of GWAS summary statistics, MR analysis cannot be stratified according to gender, race, underlying diseases, etc. We studied the causal association between CVD and TB BMD in different age groups; the results showed a strong relationship between CHD and TB BMD. But, we only observed suggestive evidence of a causal association between CHD and MI and the 0-15 population. This may be due to the small number of TB BMD samples in each age group and the fact that they come from a mixed population. In the future, more data from the same population sample size will be needed to evaluate the relationship between CVD and TB BMD in different age groups further. Fourth, OP and CVD data sources employed in Mendelian Randomization analyses of two samples should refrain from including overlapping participants. Accurate estimation poses a significant challenge. Nonetheless, the utilization of robust instrumentation has the potential to effectively mitigate sample overlap, exemplified by F statistics that markedly exceed 10.

## 5 Conclusion

The study results revealed that CHD was causally associated with Heel BMD and TB BMD, while in the reverse MR analysis, the causal relationship between OP and CVD was not supported. This result posits CHD as a potential etiological factor for OP and prompts that routine bone density assessment at traditional sites (forearm, femoral neck, lumbar spine) using DAX may inadequately discern underlying osteoporosis issues in CHD patients. The recommendation is to synergistically incorporate heel ultrasound or DAX for total body bone density examinations, ensuring clinical diagnostics are both precise and reliable. Moreover, these findings provide valuable insights for public health, contributing to the development of pertinent prevention and treatment strategies.

## Data availability statement

The original contributions presented in the study are included in the article/**Supplementary Material**. Further inquiries can be directed to the corresponding author.

## Author contributions

JZ: Writing – original draft, Writing – review & editing. PX: Writing – original draft, Writing – review & editing. RL: Writing – original draft, Writing – review & editing. JG: Writing – original draft, Writing – review & editing. PC: Writing – original draft, Writing – review & editing. CK: Supervision, Writing – original draft, Writing – review & editing.
