# Supplementary material for: Osteoporosis and coronary heart disease: a bi-directional Mendelian randomization study
Source: Front Endocrinol (Lausanne). 2024 May 22;15:1362428. doi: 10.3389/fendo.2024.1362428 (PMC11150617; doi:10.3389/fendo.2024.1362428)
Supplement: Supplementary file 2 [file DataSheet_1.doc]

，

***Supplementary Material***

**Supplementary Figure S1.**

Scatter plots of causal estimates of exposure (Cardiovascular diseases) on Forearm bone mineral density(p＜5×10^-8^). The slope of each line corresponding to the estimated MR effect in different models, including the conventional IVW, Weighted median, MR-Egger, Simple mode, and Weighted mode. (A): Coronary heart disease; (B): Myocardial infarction; (C): Stroke

**
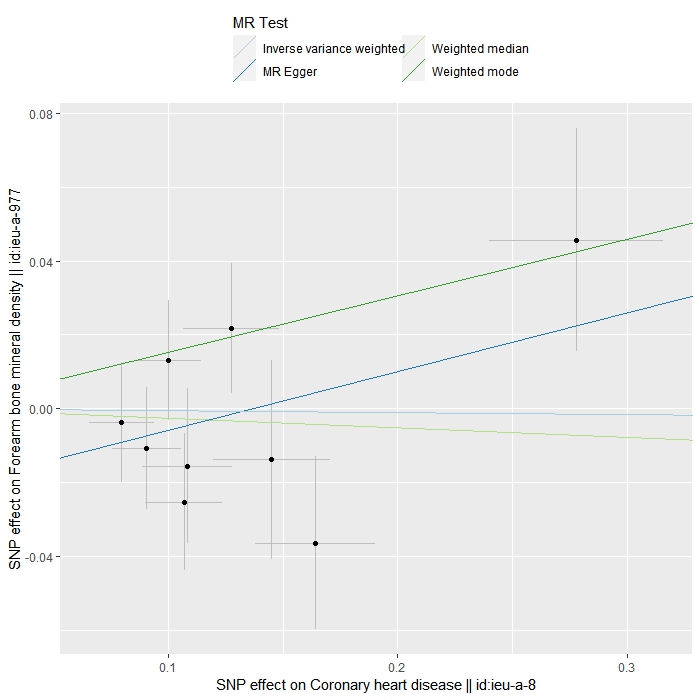

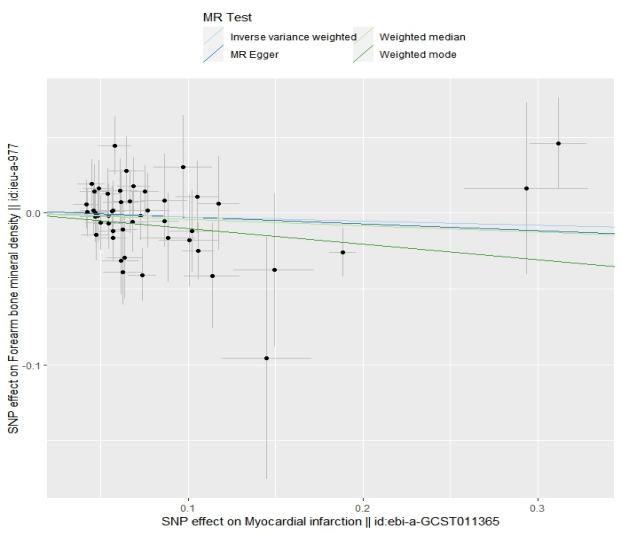
**

A B


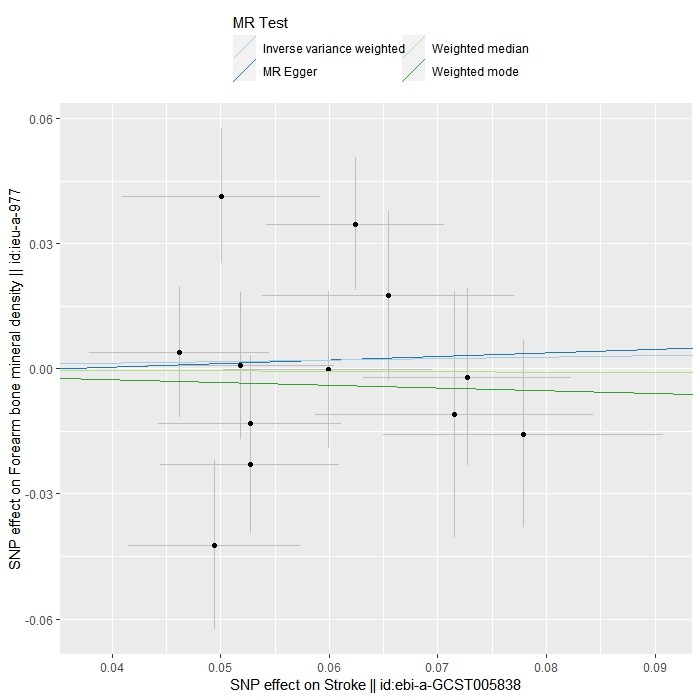


C

**Supplementary Figure S2.**

Scatter plots of causal estimates of exposure (Cardiovascular diseases) on Femoral neck mineral density. The slope of each line corresponding to the estimated MR effect in different models, including the conventional IVW, Weighted median, MR-Egger, Simple mode, and Weighted mode. (A): Coronary heart disease; (B): Myocardial infarction; (C): Stroke

**
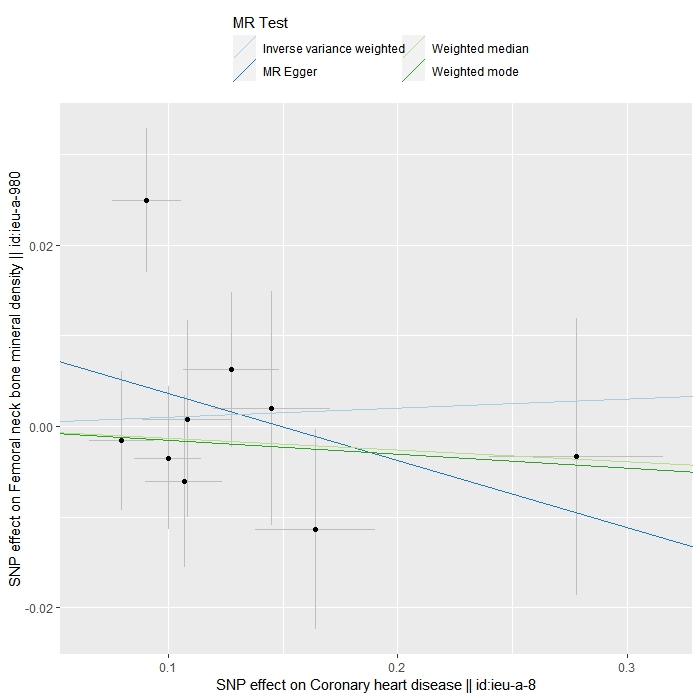
**
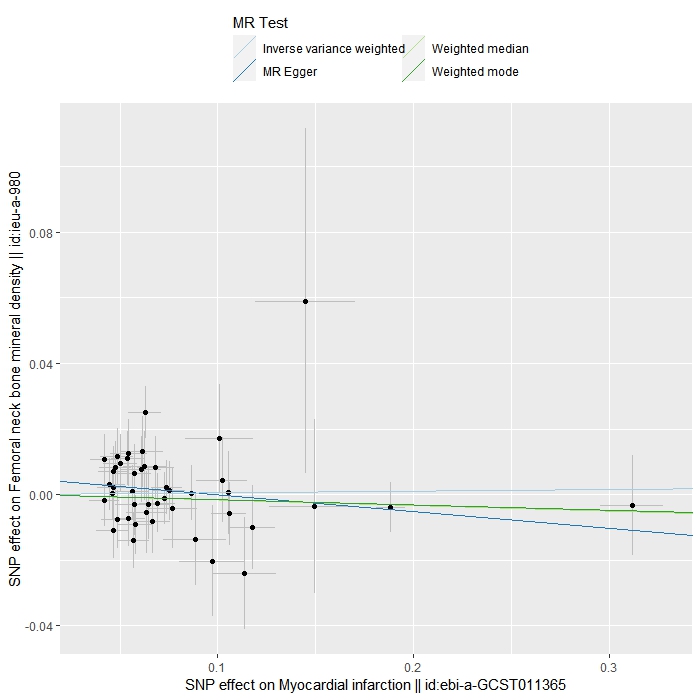


A B


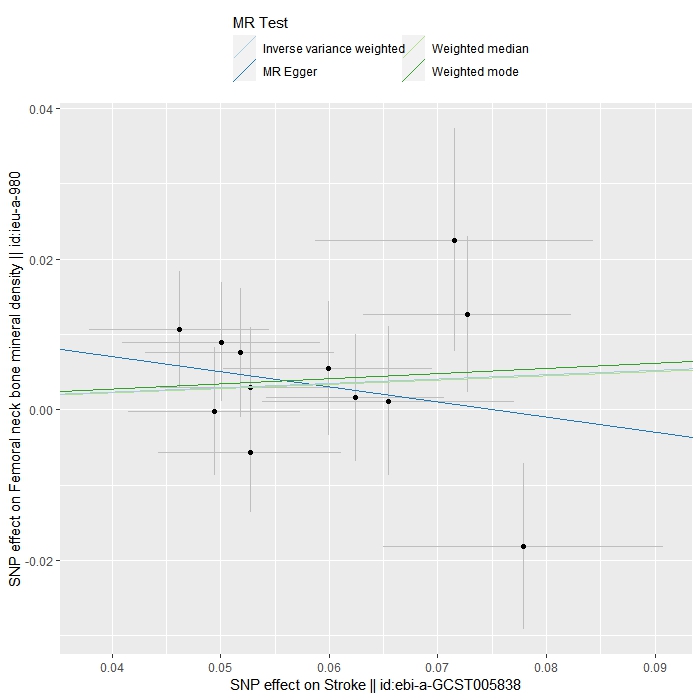


C

**Supplementary Figure S3.**

Scatter plots of causal estimates of exposure (Cardiovascular diseases) on Heel bone mineral density. The slope of each line corresponding to the estimated MR effect in different models, including the conventional IVW, Weighted median, MR-Egger, Simple mode, and Weighted mode. (A): Coronary heart disease; (B): Myocardial infarction; (C): Stroke

**
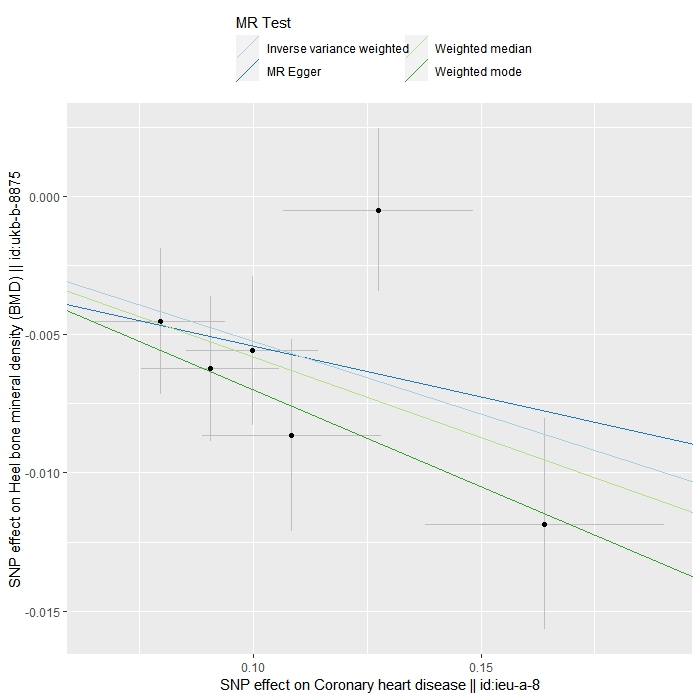
**
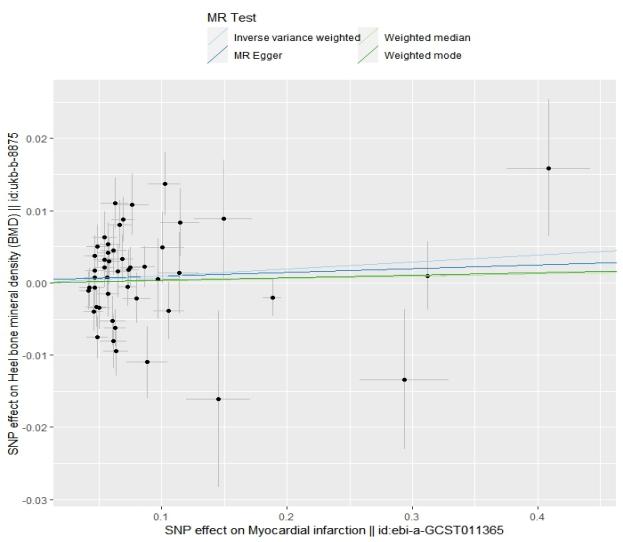


A B


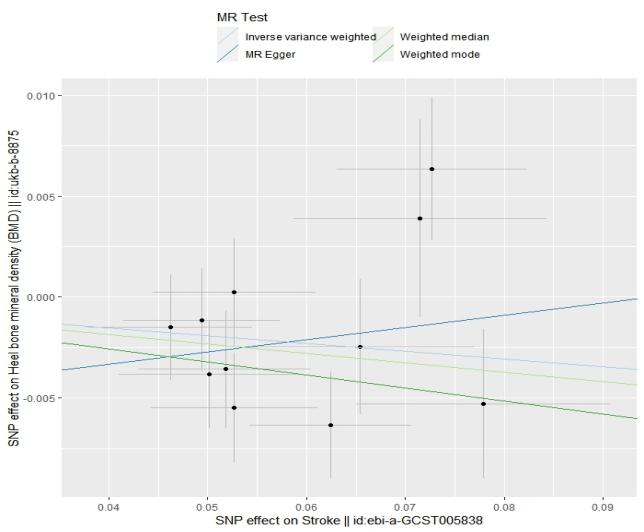


C

**Supplementary Figure S4.**

Scatter plots of causal estimates of exposure (Cardiovascular diseases) on Lumbar spine bone density. The slope of each line corresponding to the estimated MR effect in different models, including the conventional IVW, Weighted median, MR-Egger, Simple mode, and Weighted mode. (A): Coronary heart disease; (B): Myocardial infarction; (C): Stroke

**
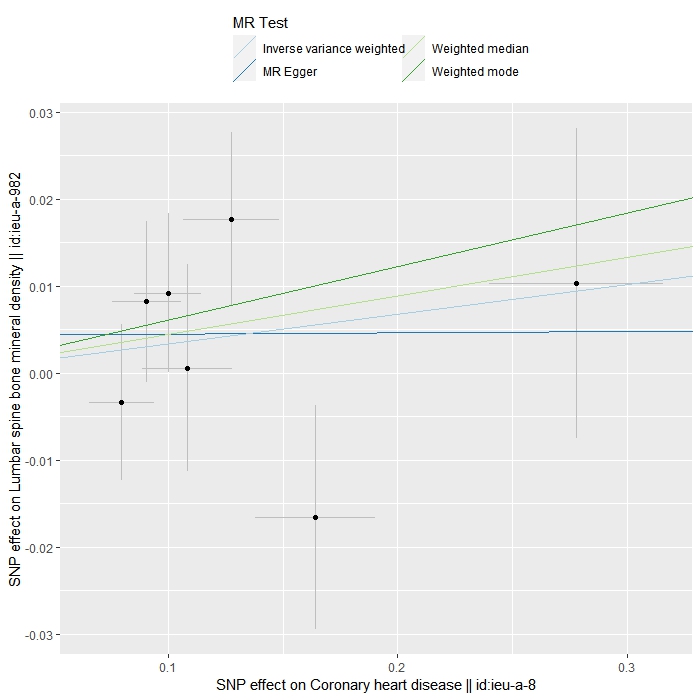
**
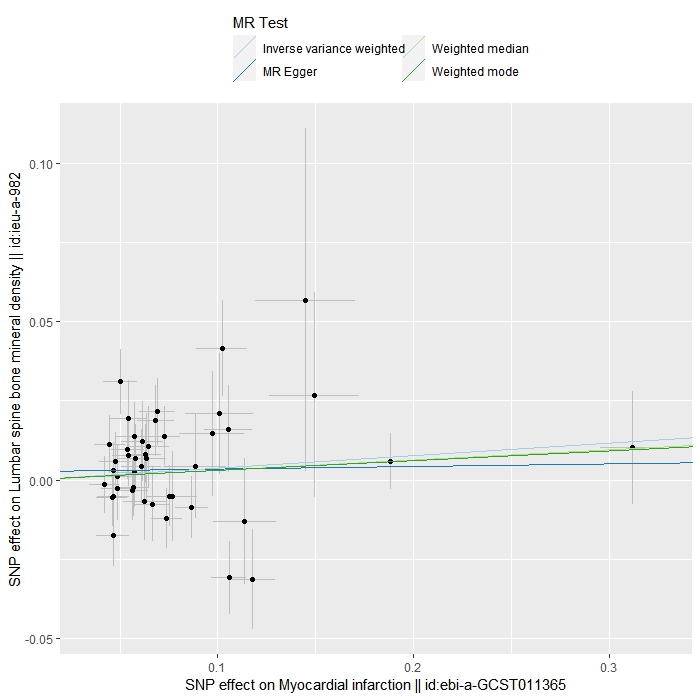


A B


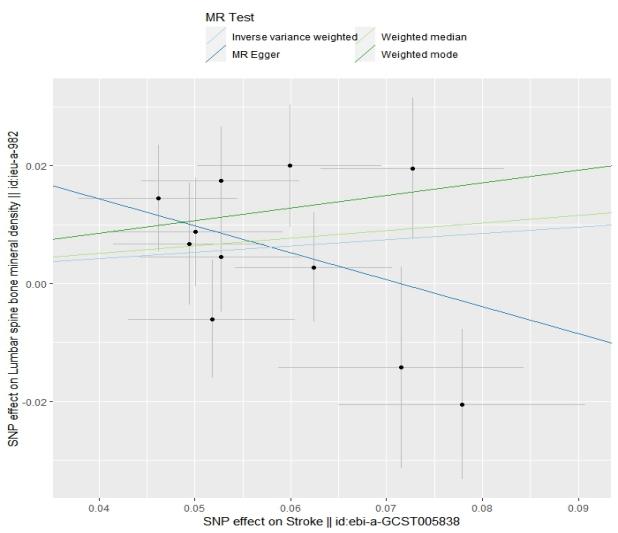


C

**Supplementary Figure S5.**

Scatter plots of causal estimates of exposure (Cardiovascular diseases) on Total body bone mineral density. The slope of each line corresponding to the estimated MR effect in different models, including the conventional IVW, Weighted median, MR-Egger, Simple mode, and Weighted mode. (A): Coronary heart disease; (B): Myocardial infarction; (C): Stroke


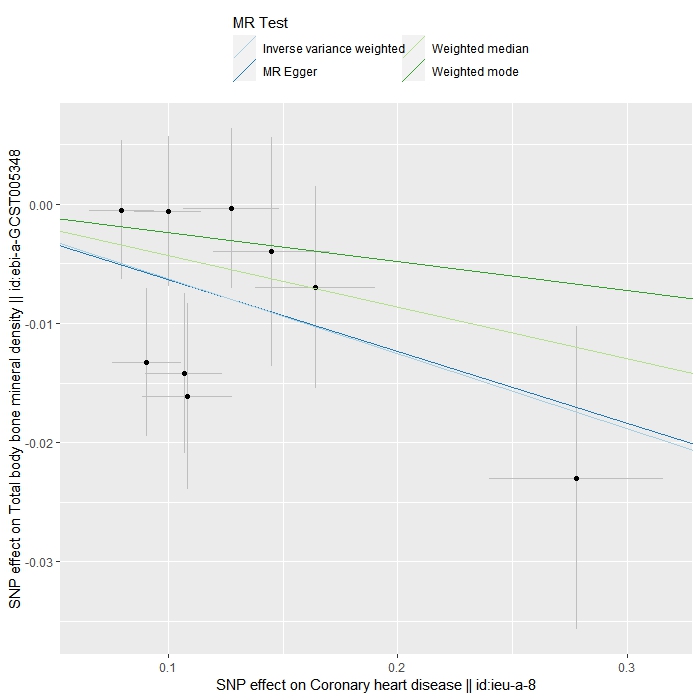

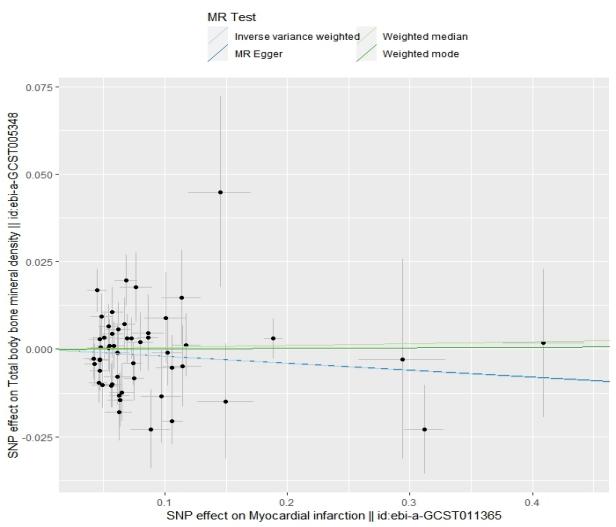


A B


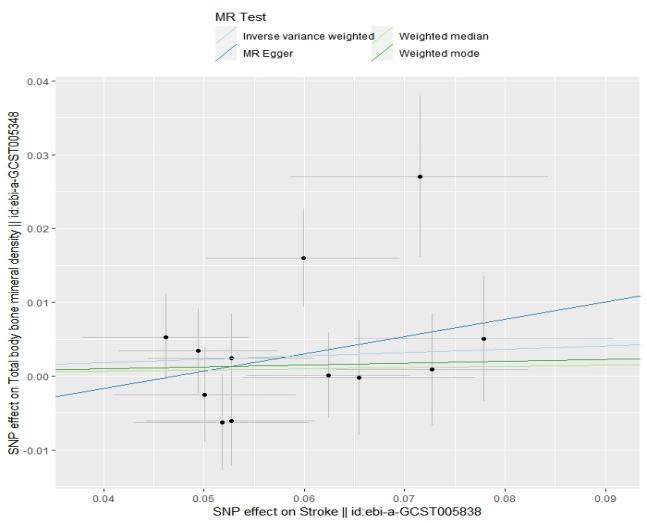


C

**Supplementary Figure S6.**

Leave-one-out stability tests causal estimates of exposure (Cardiovascular diseases) on Forearm bone mineral density(p＜5×10^-8^). The slope of each line corresponding to the estimated MR effect in different models, including the conventional IVW, Weighted median, MR-Egger, Simple mode, and Weighted mode. (A): coronary heart disease; (B): myocardial infarction; (C): Stroke


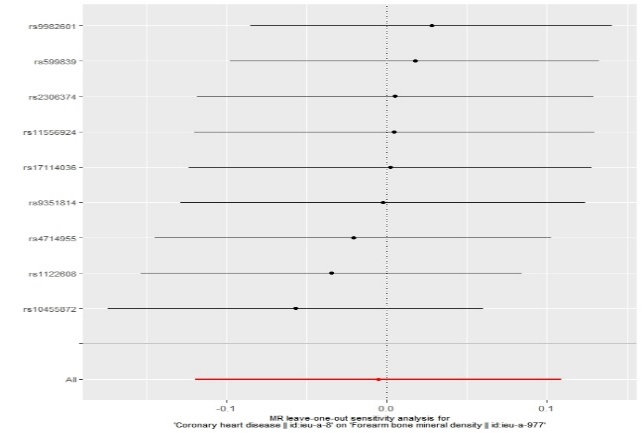

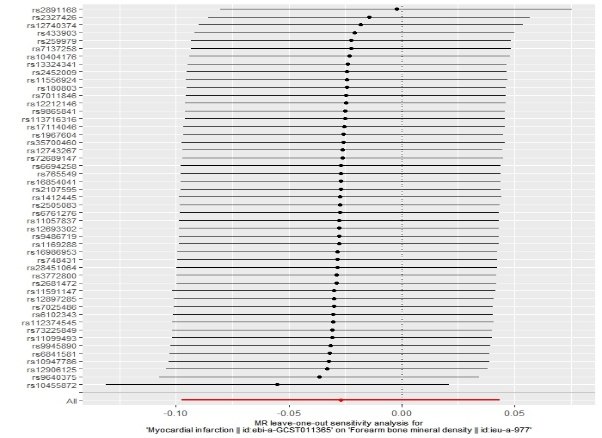


A B


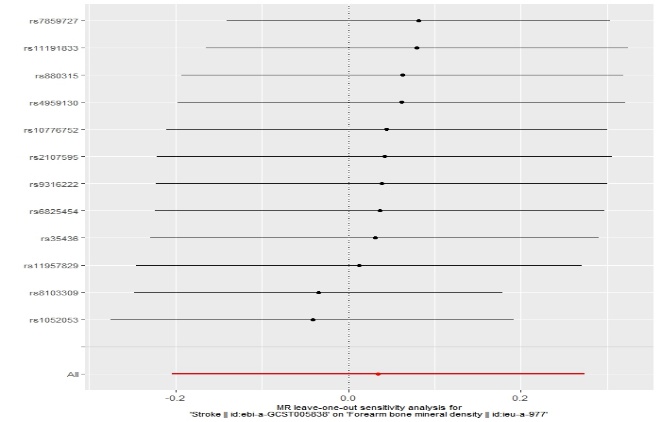


C

**Supplementary Figure S7.**

Leave-one-out stability tests causal estimates of exposure (Cardiovascular diseases) on Femoral neck mineral density. The slope of each line corresponding to the estimated MR effect in different models, including the conventional IVW, Weighted median, MR-Egger, Simple mode, and Weighted mode. (A): coronary heart disease; (B): myocardial infarction; (C): Stroke


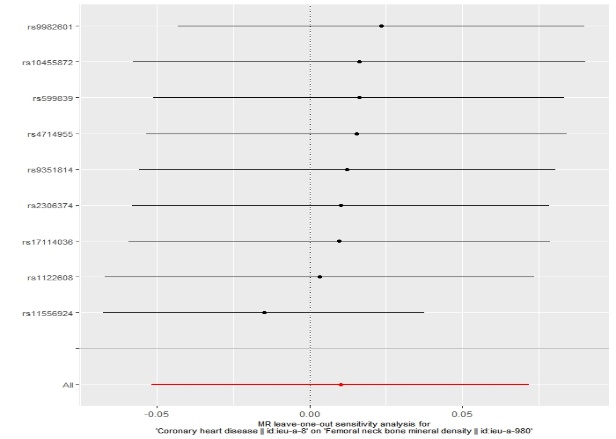

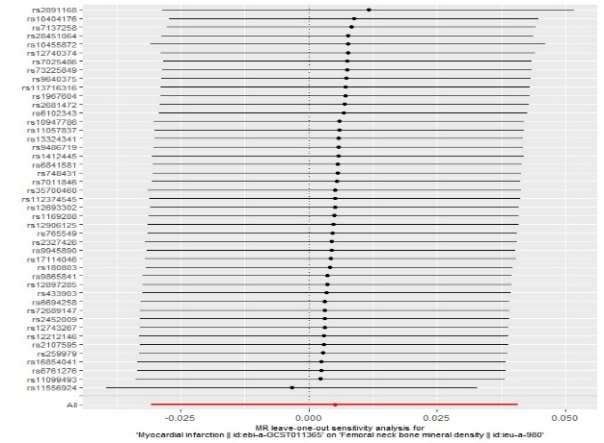


A B


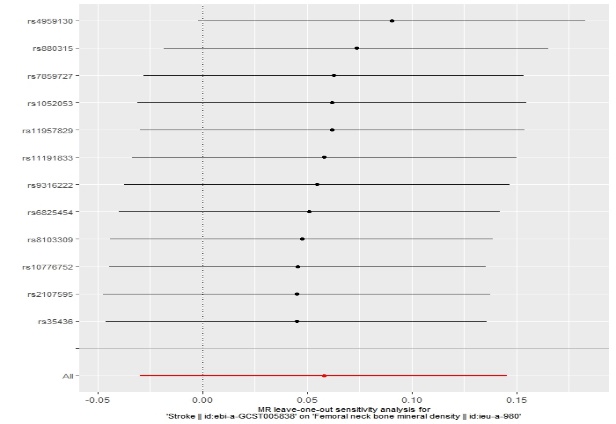


C

**Supplementary Figure S8.**

Leave-one-out stability tests causal estimates of exposure (Cardiovascular diseases) on Heel bone mineral density. The slope of each line corresponding to the estimated MR effect in different models, including the conventional IVW, Weighted median, MR-Egger, Simple mode, and Weighted mode. (A): coronary heart disease; (B): myocardial infarction; (C): Stroke


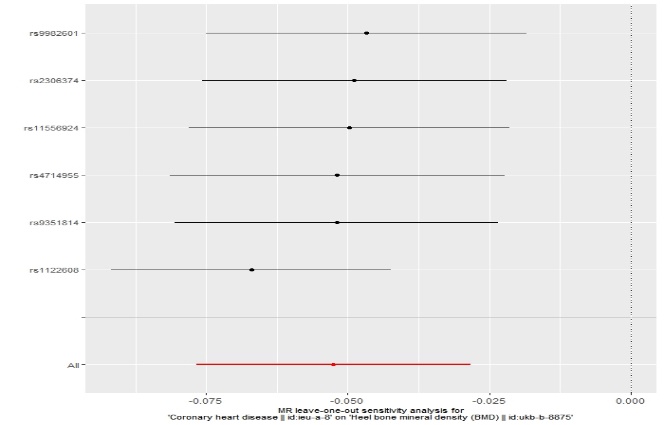

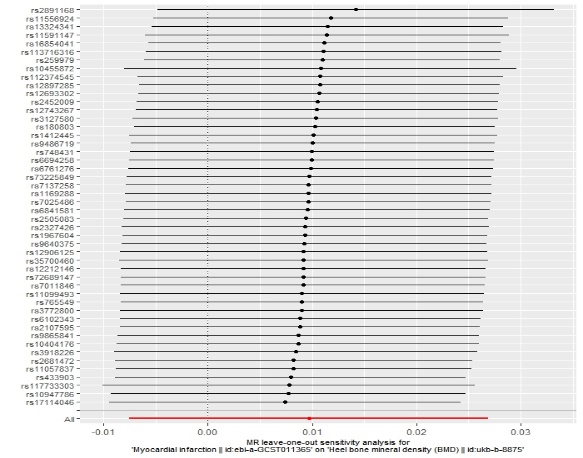
A B


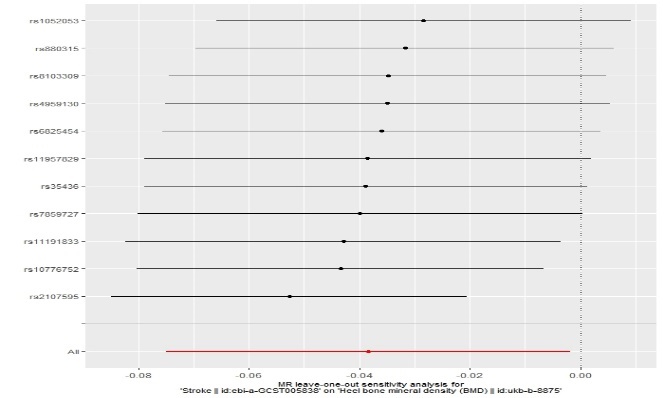


C

**Supplementary Figure S9.**

Leave-one-out stability tests causal estimates of exposure (Cardiovascular diseases) on Lumbar spine bone density. The slope of each line corresponding to the estimated MR effect in different models, including the conventional IVW, Weighted median, MR-Egger, Simple mode, and Weighted mode. (A): coronary heart disease; (B): myocardial infarction; (C): Stroke


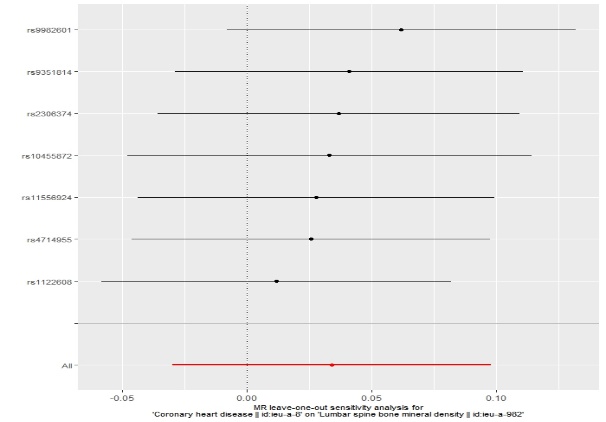

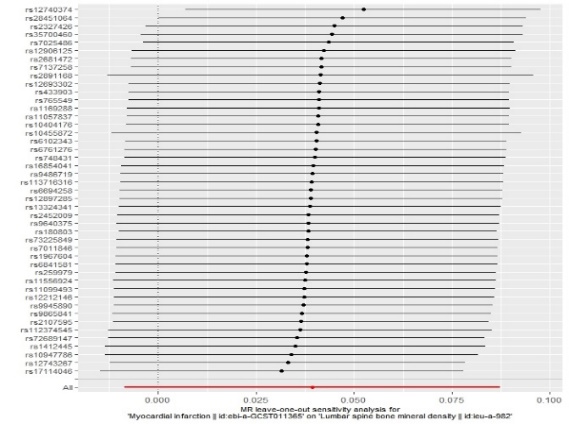


A B


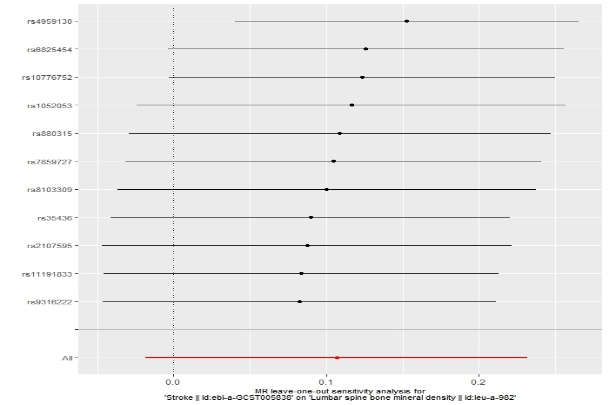


C

**Supplementary Figure S10.**

Leave-one-out stability tests causal estimates of exposure (Cardiovascular diseases) on Total body bone mineral density. The slope of each line corresponding to the estimated MR effect in different models, including the conventional IVW, Weighted median, MR-Egger, Simple mode, and Weighted mode. (A): coronary heart disease; (B): myocardial infarction; (C): Stroke


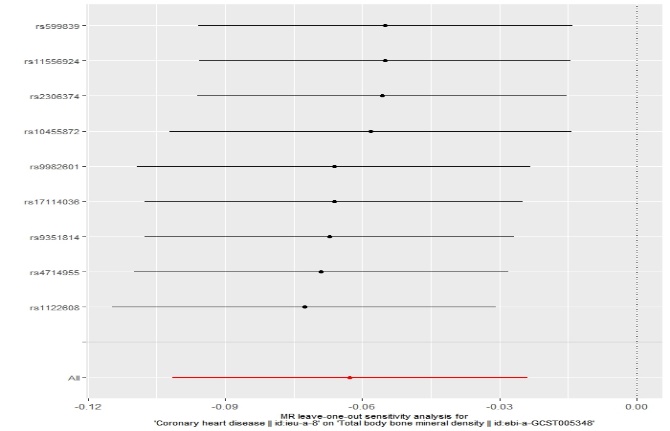

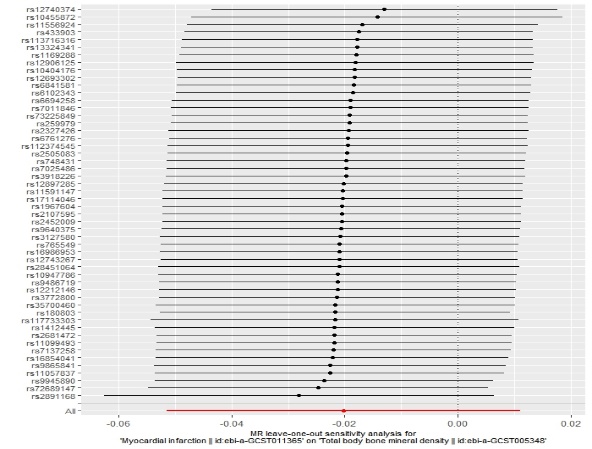


A B


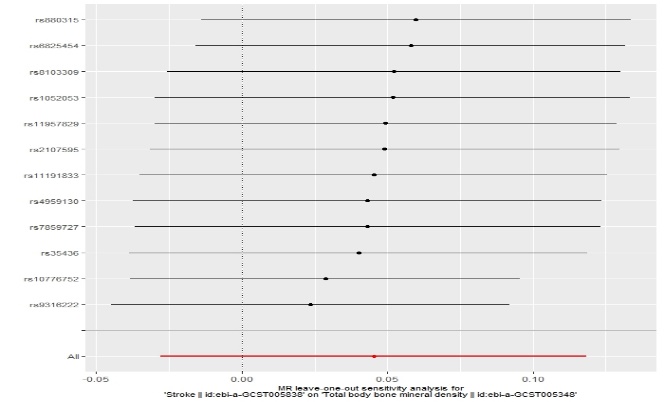


C

**Supplementary Figure S11.**

Forest plots of causal estimates of exposure (Cardiovascular diseases) on Forearm bone mineral density(p＜5×10^-8^). The slope of each line corresponding to the estimated MR effect in different models, including the conventional IVW, Weighted median, MR-Egger, Simple mode, and Weighted mode. (A): coronary heart disease; (B): myocardial infarction; (C): Stroke


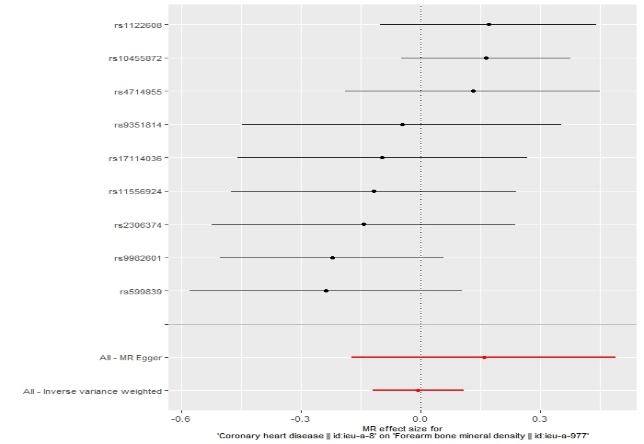

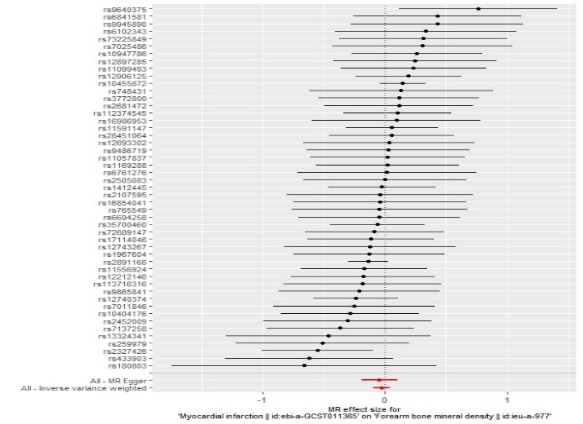


A B


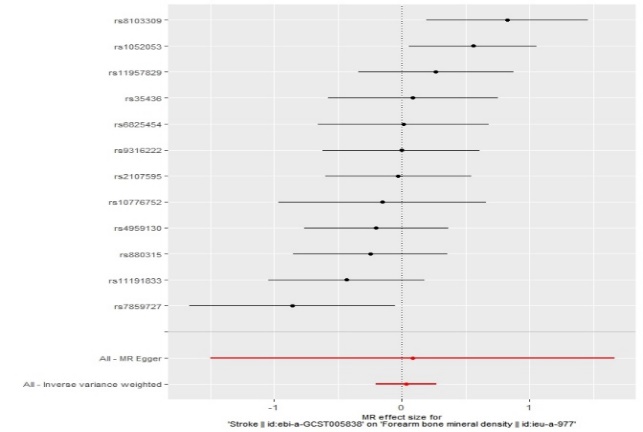


C

**Supplementary Figure S12.**

Forest plots of causal estimates of exposure (Cardiovascular diseases) on Femoral neck mineral density. The slope of each line corresponding to the estimated MR effect in different models, including the conventional IVW, Weighted median, MR-Egger, Simple mode, and Weighted mode. (A): coronary heart disease; (B): myocardial infarction; (C): Stroke


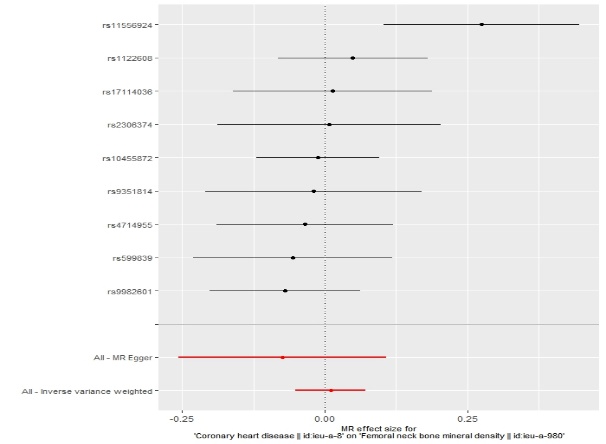

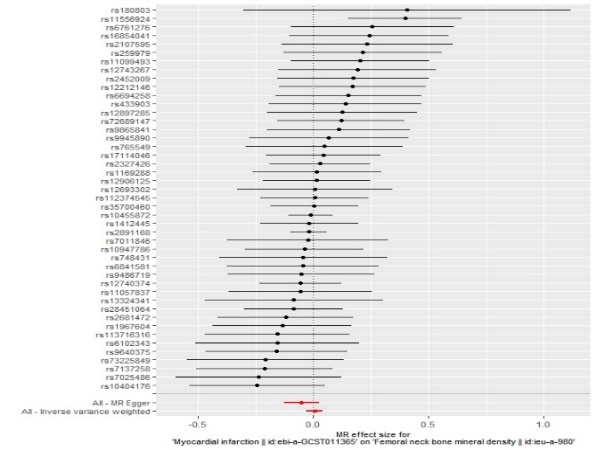


A B


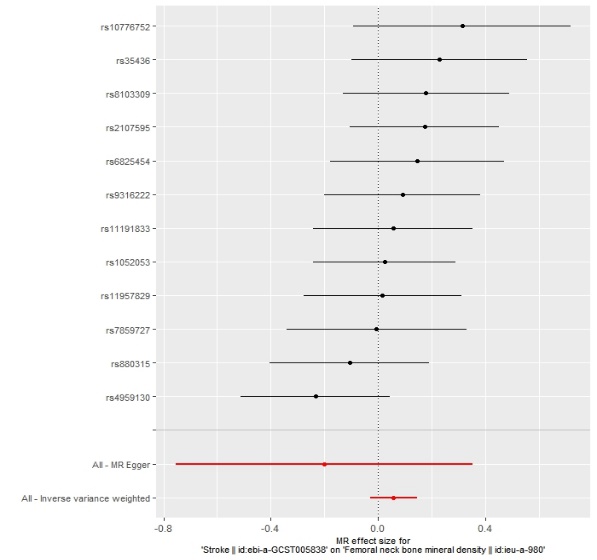


C

**Supplementary Figure S13.**

Forest plots of causal estimates of exposure (Cardiovascular diseases) on Heel bone mineral density. The slope of each line corresponding to the estimated MR effect in different models, including the conventional IVW, Weighted median, MR-Egger, Simple mode, and Weighted mode. (A): coronary heart disease; (B): myocardial infarction; (C): Stroke


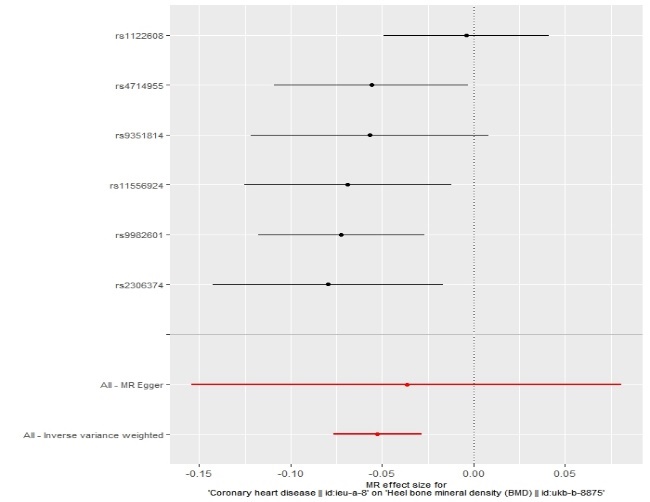

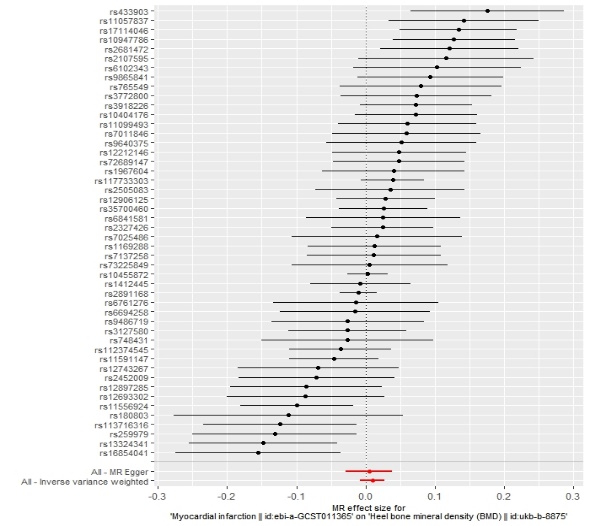


A B


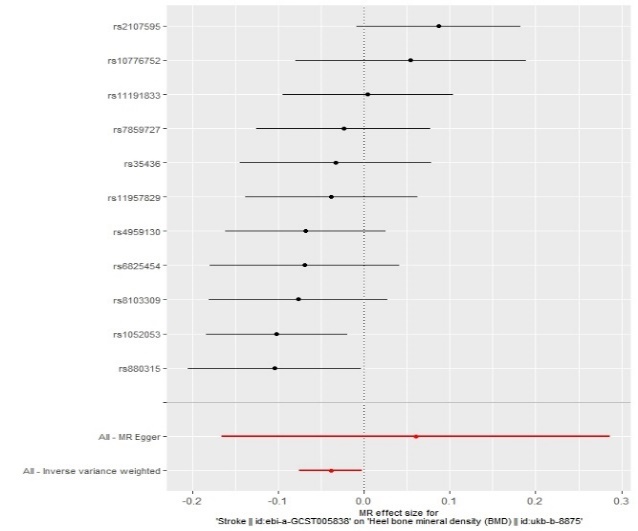


C

**Supplementary Figure S14.**

Forest plots of causal estimates of exposure (Cardiovascular diseases) on Lumbar spine bone density. The slope of each line corresponding to the estimated MR effect in different models, including the conventional IVW, Weighted median, MR-Egger, Simple mode, and Weighted mode. (A): coronary heart disease; (B): myocardial infarction; (C): Stroke


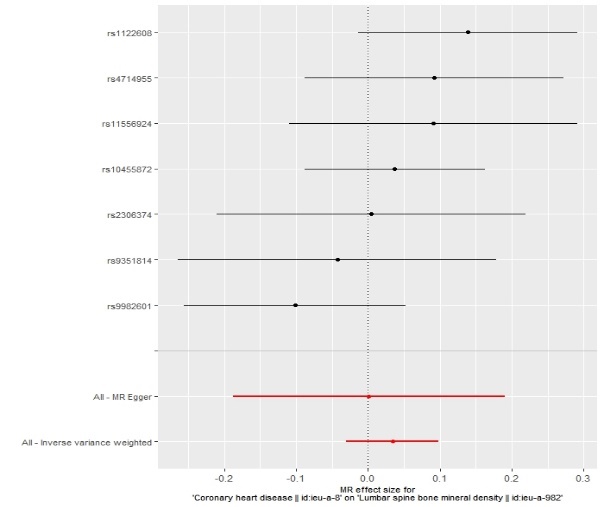

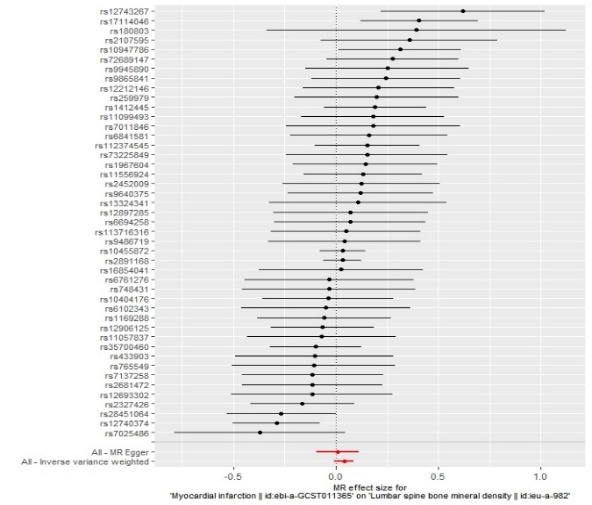


A B


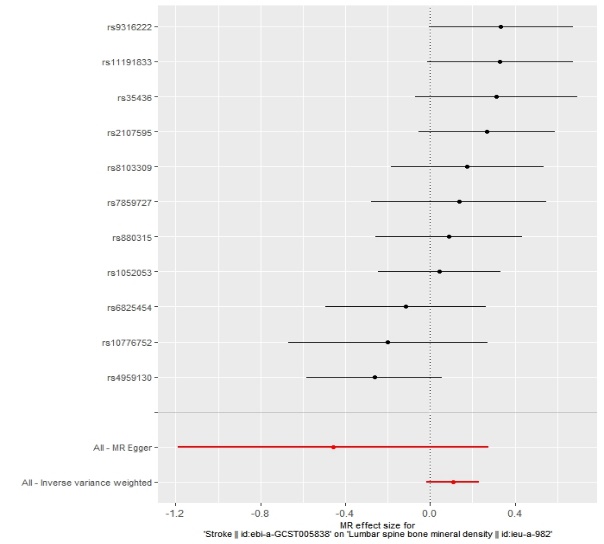


C

**Supplementary Figure S15.**

Forest plots of causal estimates of exposure (Cardiovascular diseases) on Total body bone mineral density. The slope of each line corresponding to the estimated MR effect in different models, including the conventional IVW, Weighted median, MR-Egger, Simple mode, and Weighted mode. (A): coronary heart disease; (B): myocardial infarction; (C): Stroke


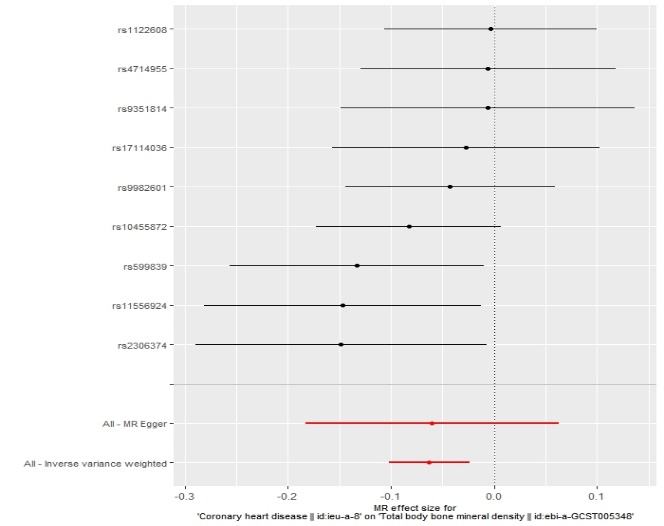

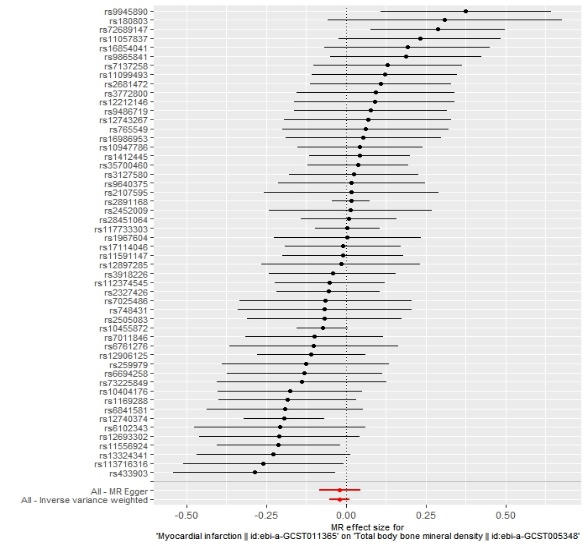


A B


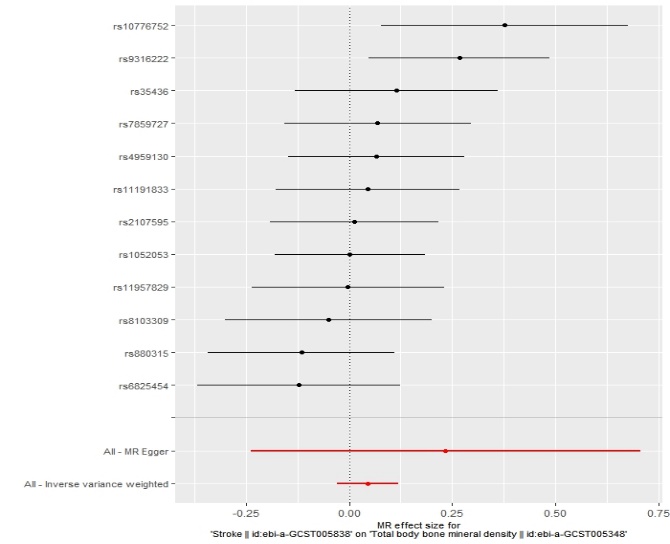


C

**Supplementary Figure S16.**

Funnel plots of causal estimates of exposure (Cardiovascular diseases) on Forearm bone mineral density(p＜5×10^-8^). The slope of each line corresponding to the estimated MR effect in different models, including the conventional IVW, Weighted median, MR-Egger, Simple mode, and Weighted mode. (A): coronary heart disease; (B): myocardial infarction; (C): Stroke


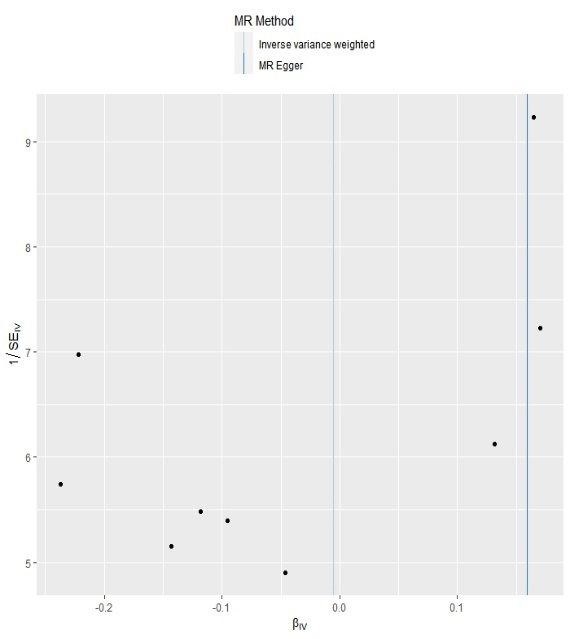

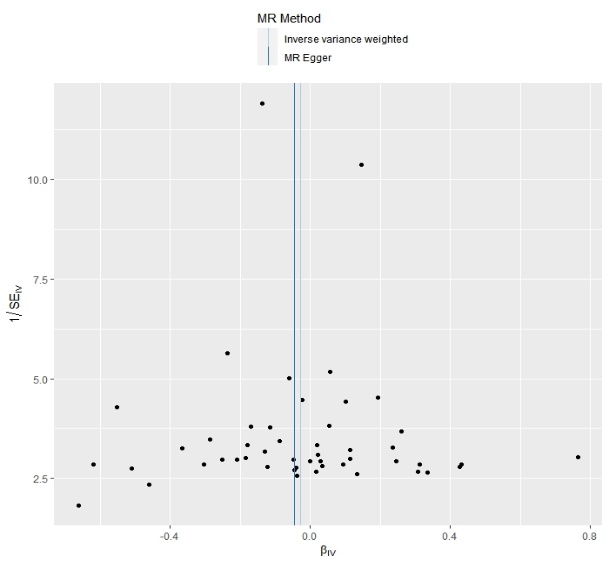
 MR effect size of for MR effect size of for

“Coronary heart disease” on “Total body bone mineral density” “Myocardial infarction” on “Forearm bone mineral density”

A B


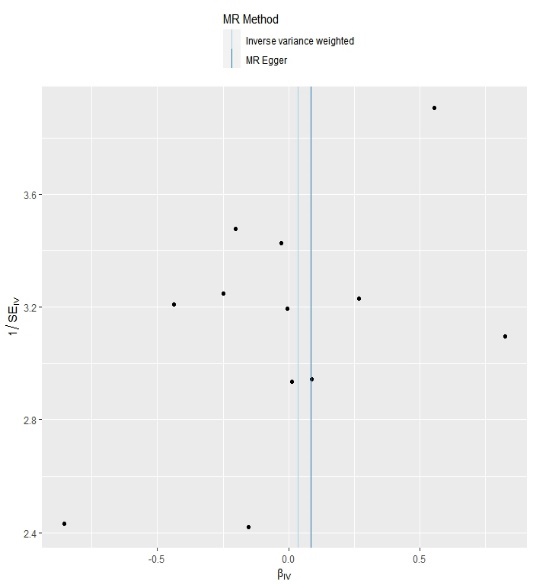


MR effect size of for

“Stroke” on “Forearm bone mineral density”

C

**Supplementary Figure S17.**

Funnel plots of causal estimates of exposure (Cardiovascular diseases) on Femoral neck mineral density. The slope of each line corresponding to the estimated MR effect in different models, including the conventional IVW, Weighted median, MR-Egger, Simple mode, and Weighted mode. (A): coronary heart disease; (B): myocardial infarction; (C): Stroke


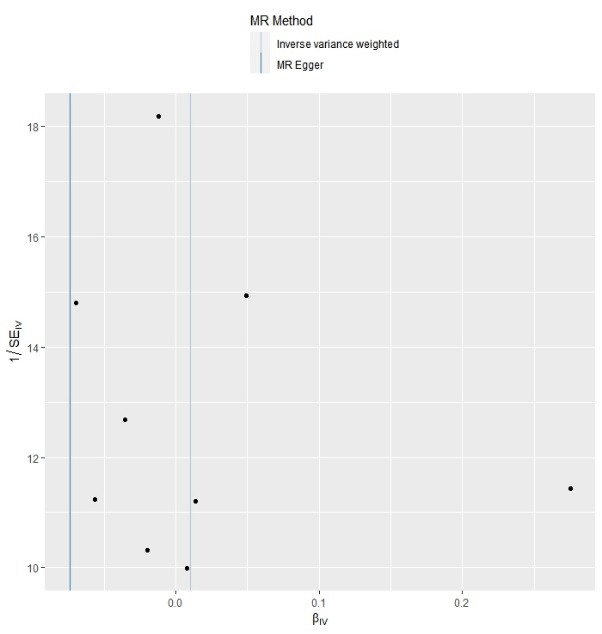

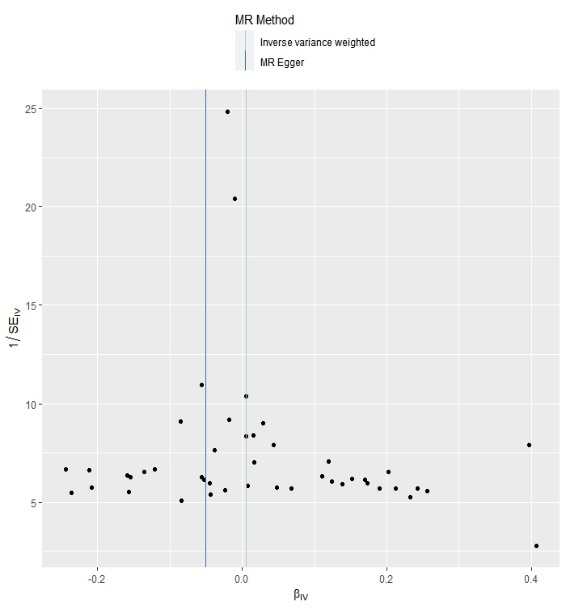


MR effect size of for MR effect size of for

“Coronary heart disease” on “Femoral neck mineral density” “Myocardial infarction” on “Femoral neck mineral density”

A B


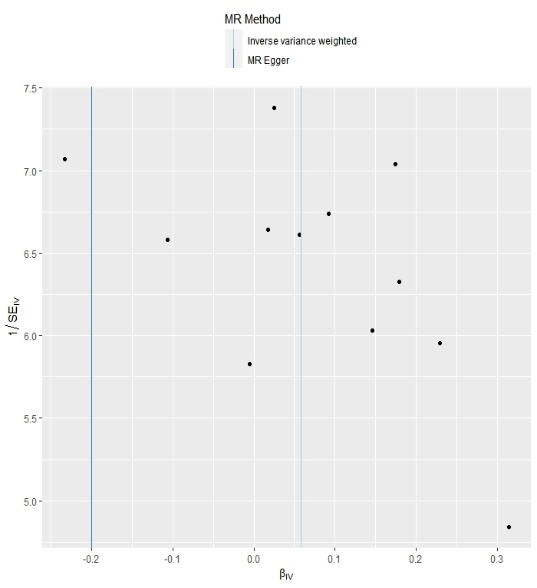


MR effect size of for

“Stroke” on “Femoral neck mineral density”

C

**Supplementary Figure S18.**

Funnel plots of causal estimates of exposure (Cardiovascular diseases) on Heel bone mineral density. The slope of each line corresponding to the estimated MR effect in different models, including the conventional IVW, Weighted median, MR-Egger, Simple mode, and Weighted mode. (A): coronary heart disease; (B): myocardial infarction; (C): Stroke


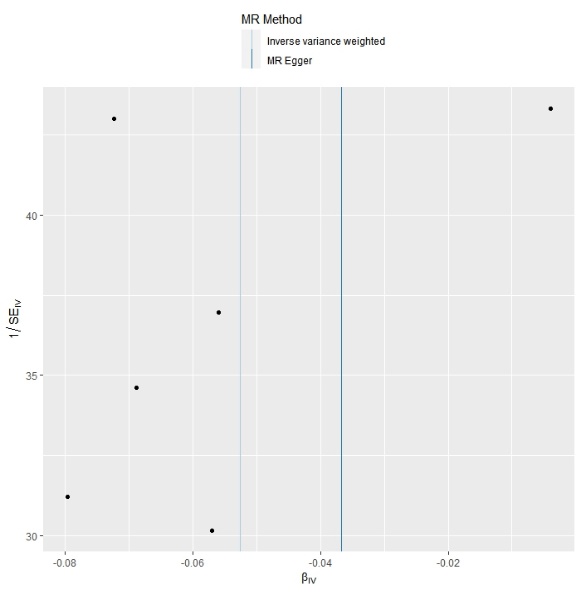

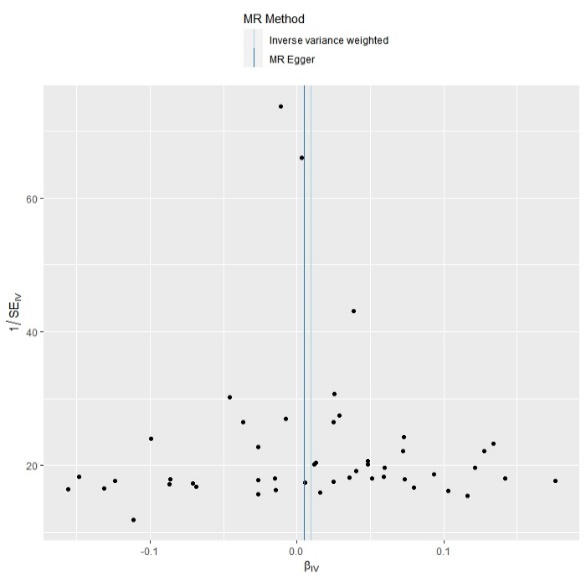


MR effect size of for MR effect size of for

“Coronary heart disease” on “Heel bone mineral density” “Myocardial infarction” on “Heel bone mineral density”

A B


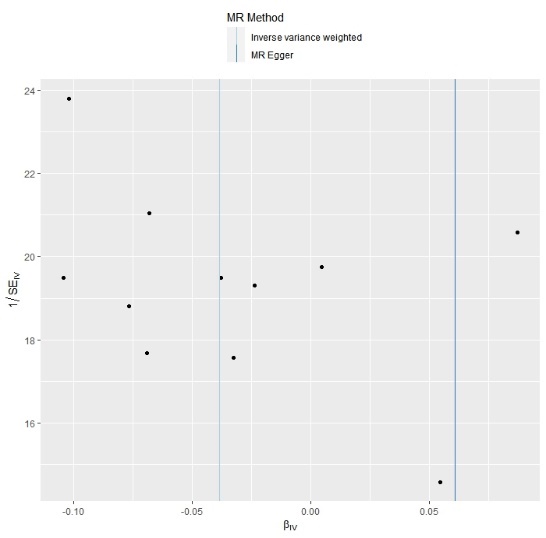


MR effect size of for

“Stroke” on “Heel bone mineral density”

C

**Supplementary Figure S19.**

Funnel plots of causal estimates of exposure (Cardiovascular diseases) on Lumbar spine bone density. The slope of each line corresponding to the estimated MR effect in different models, including the conventional IVW, Weighted median, MR-Egger, Simple mode, and Weighted mode. (A): coronary heart disease; (B): myocardial infarction; (C): Stroke


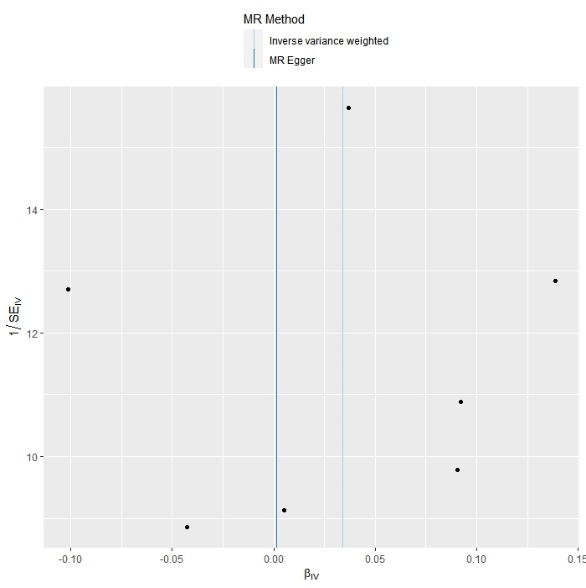

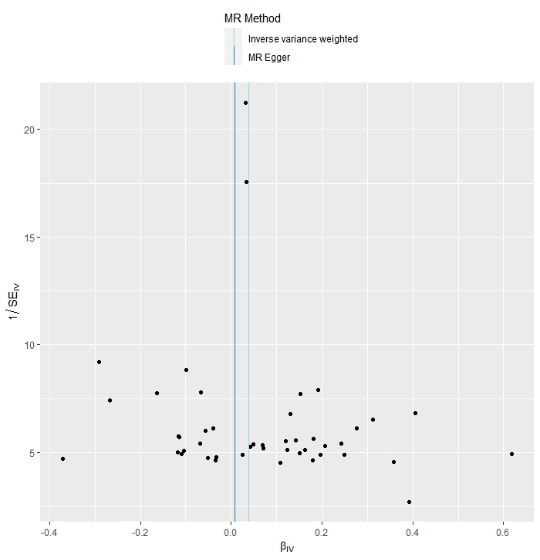


MR effect size of for MR effect size of for

“Coronary heart disease” on “Lumbar spine bone density” “Myocardial infarction” on “Lumbar spine bone density”

A B


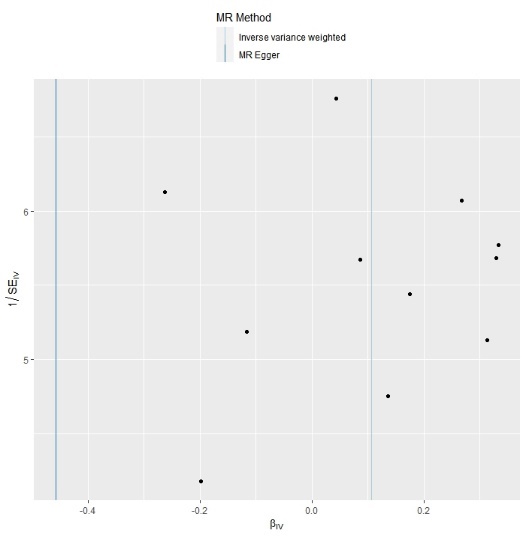


MR effect size of for

“Stroke” on “Lumbar spine bone density”

C

**Supplementary Figure S20.**

Funnel plots of causal estimates of exposure (Cardiovascular diseases) on Total body bone mineral density. The slope of each line corresponding to the estimated MR effect in different models, including the conventional IVW, Weighted median, MR-Egger, Simple mode, and Weighted mode. (A): coronary heart disease; (B): myocardial infarction; (C): Stroke


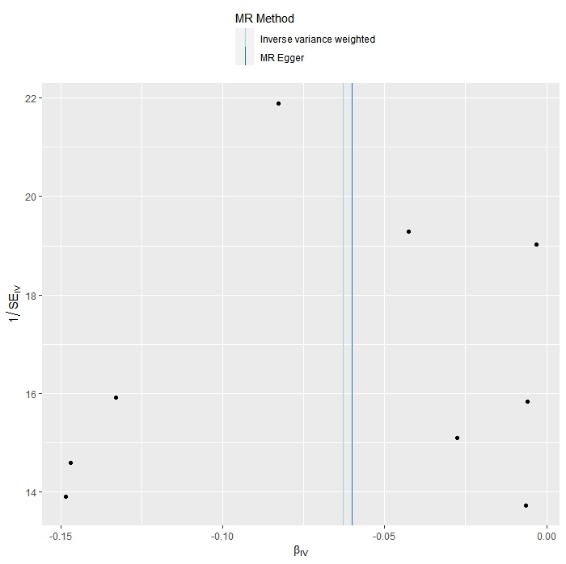

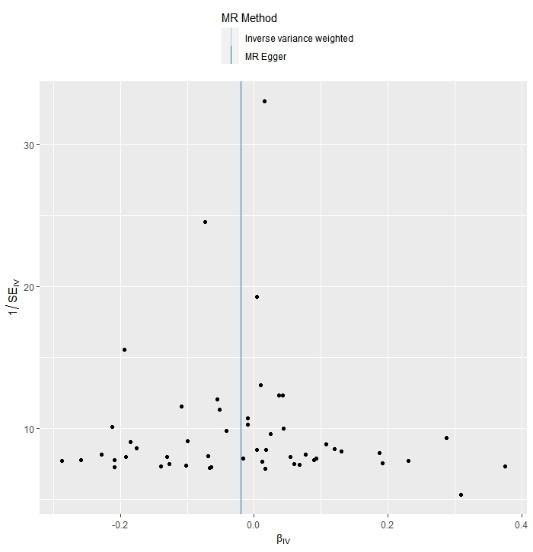


MR effect size of for MR effect size of for

“Coronary heart disease” on “Total body bone mineral density” “Myocardial infarction” on “Total body bone mineral density”

A B


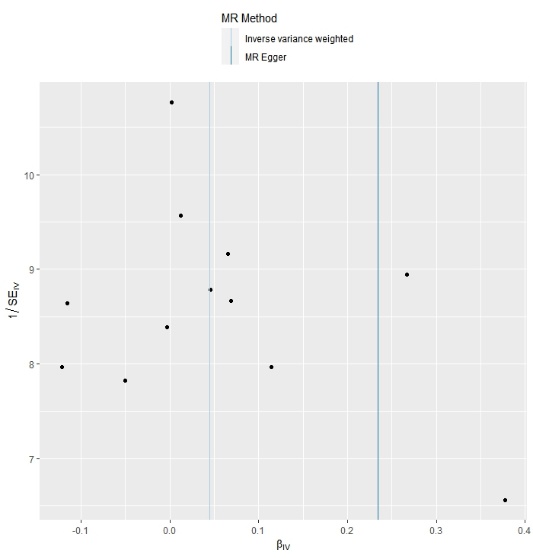


MR effect size of for

“Stroke” on “Total body bone mineral density”

**Supplementary Figure S21.**

Scatter plots of causal estimates of exposure (Cardiovascular diseases) on Total body bone mineral density (age0-15). The slope of each line corresponding to the estimated MR effect in different models, including the conventional IVW, Weighted median, MR-Egger, Simple mode, and Weighted mode. (A): Coronary heart disease; (B): Myocardial infarction; (C): Stroke


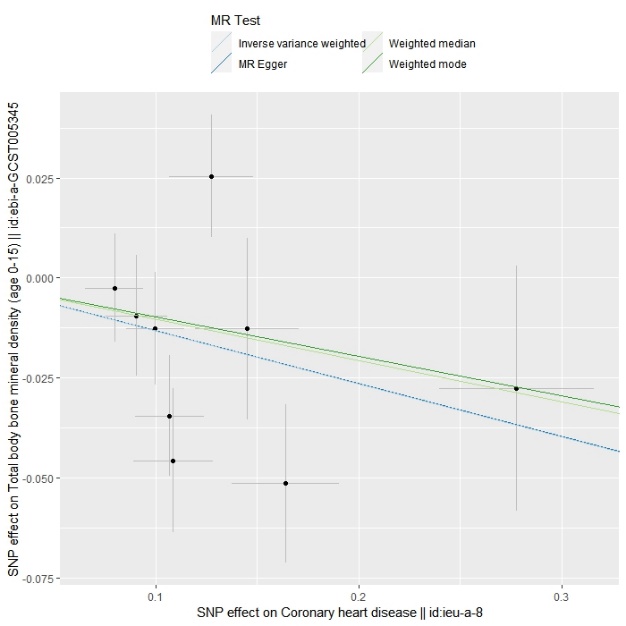

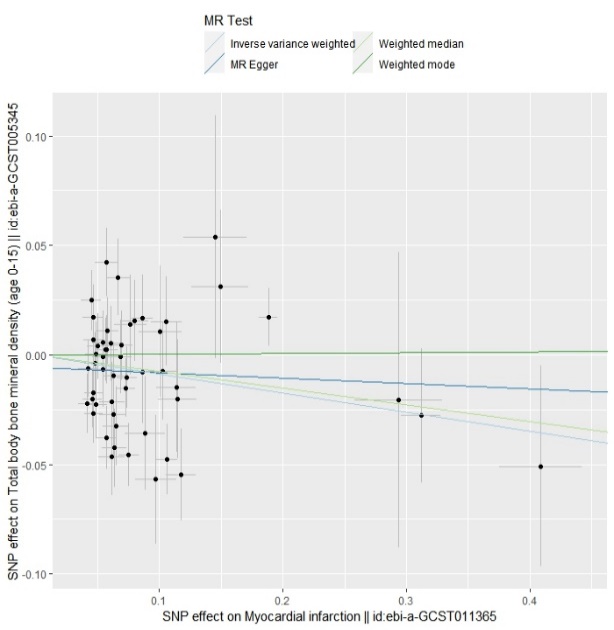


A B


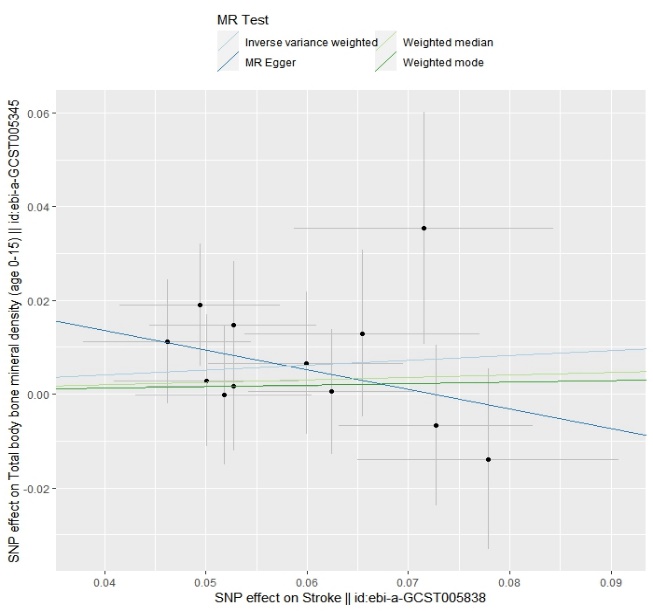


C

**Supplementary Figure S22.**

Scatter plots of causal estimates of exposure (Cardiovascular diseases) on Total body bone mineral density (age15-30). The slope of each line corresponding to the estimated MR effect in different models, including the conventional IVW, Weighted median, MR-Egger, Simple mode, and Weighted mode. (A): Coronary heart disease; (B): Myocardial infarction; (C): Stroke


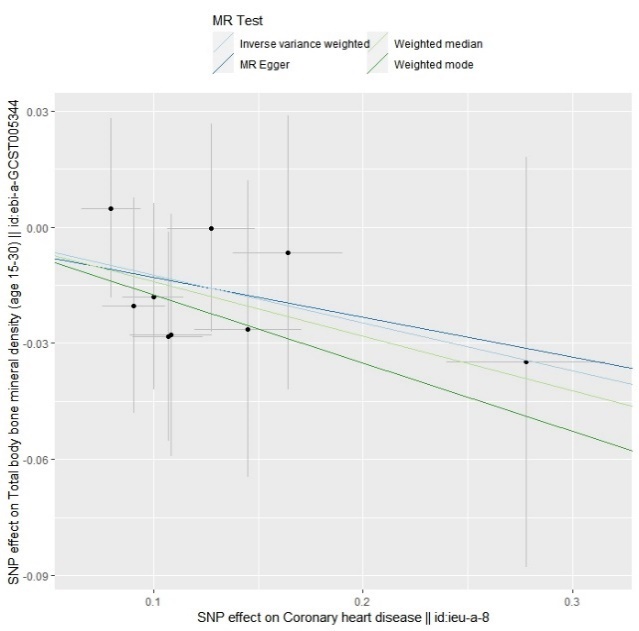

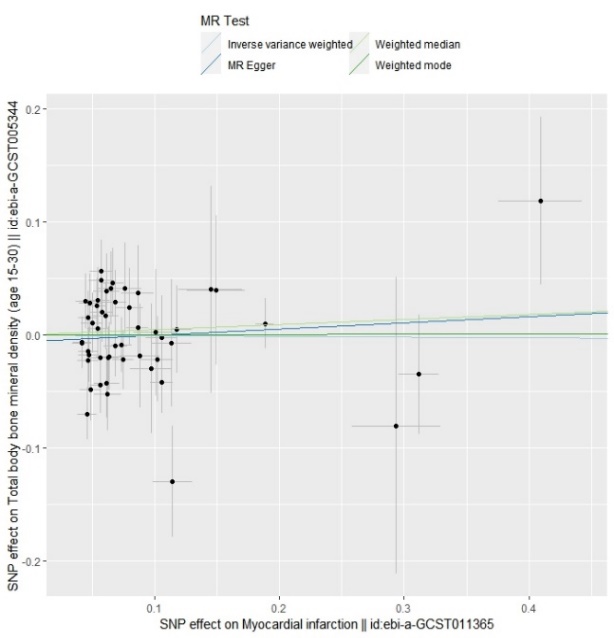


A B


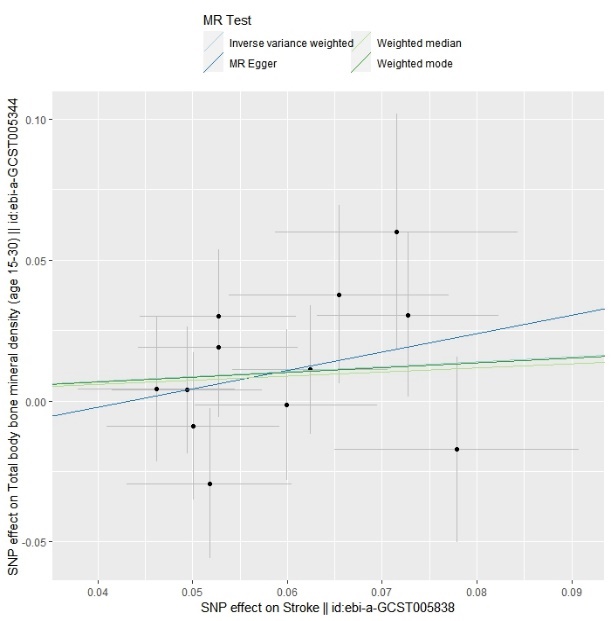


C

**Supplementary Figure S23.**

Scatter plots of causal estimates of exposure (Cardiovascular diseases) on Total body bone mineral density (age30-45). The slope of each line corresponding to the estimated MR effect in different models, including the conventional IVW, Weighted median, MR-Egger, Simple mode, and Weighted mode. (A): Coronary heart disease; (B): Myocardial infarction; (C): Stroke


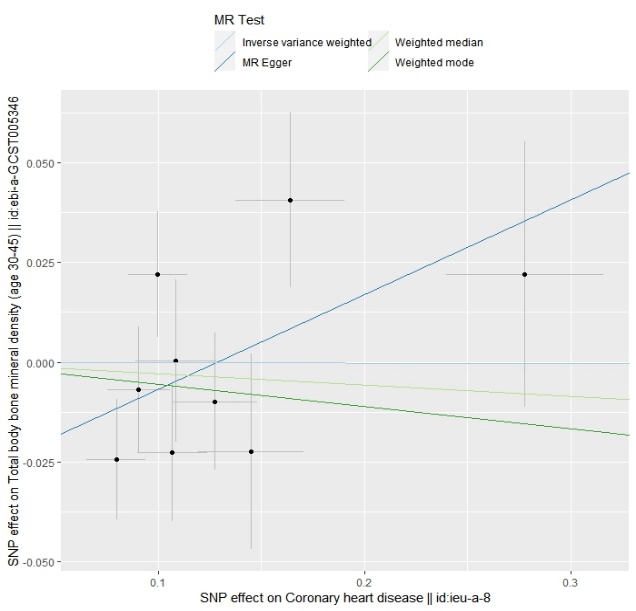

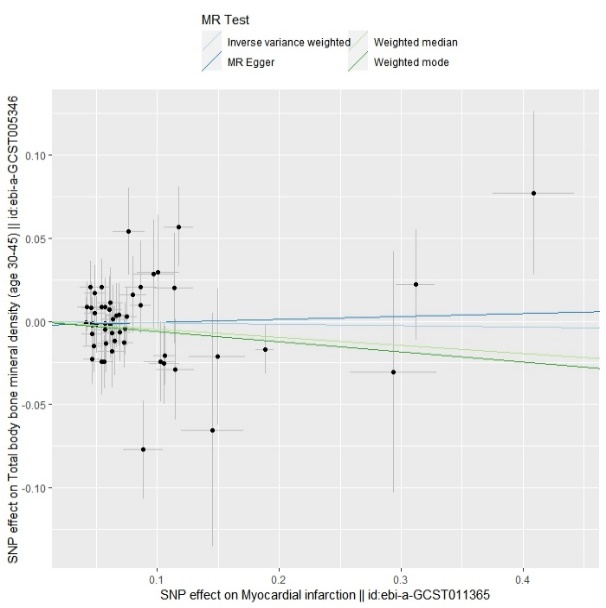


A B


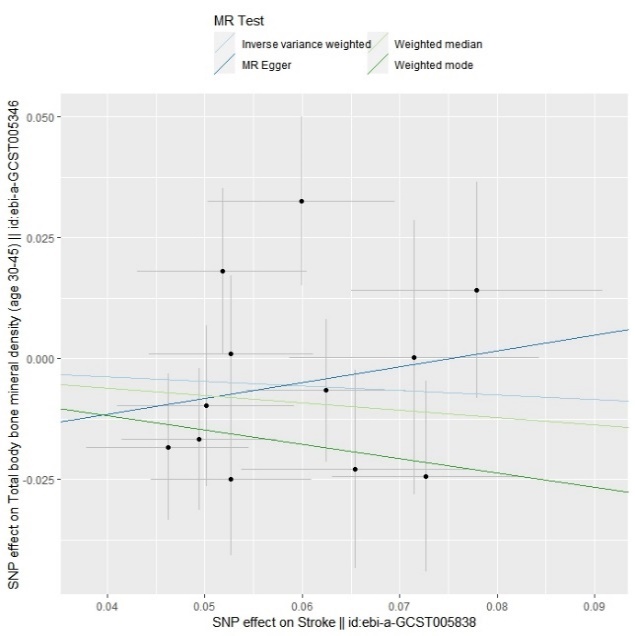


C

**Supplementary Figure S24.**

Scatter plots of causal estimates of exposure (Cardiovascular diseases) on Total body bone mineral density (age45-60). The slope of each line corresponding to the estimated MR effect in different models, including the conventional IVW, Weighted median, MR-Egger, Simple mode, and Weighted mode. (A): Coronary heart disease; (B): Myocardial infarction; (C): Stroke


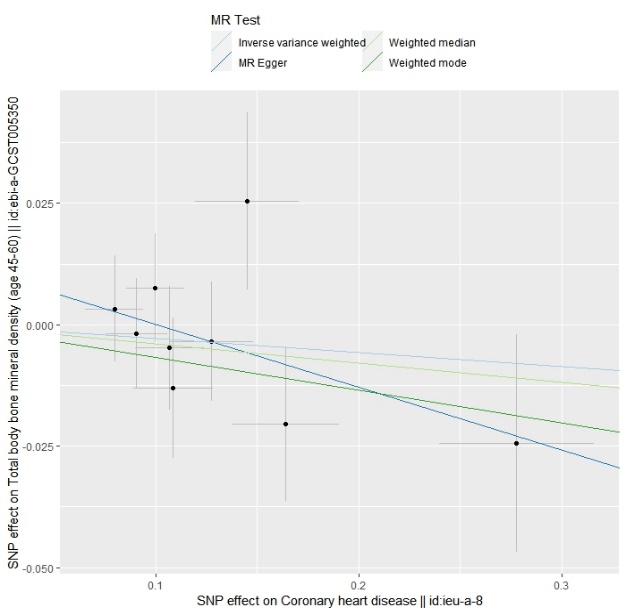

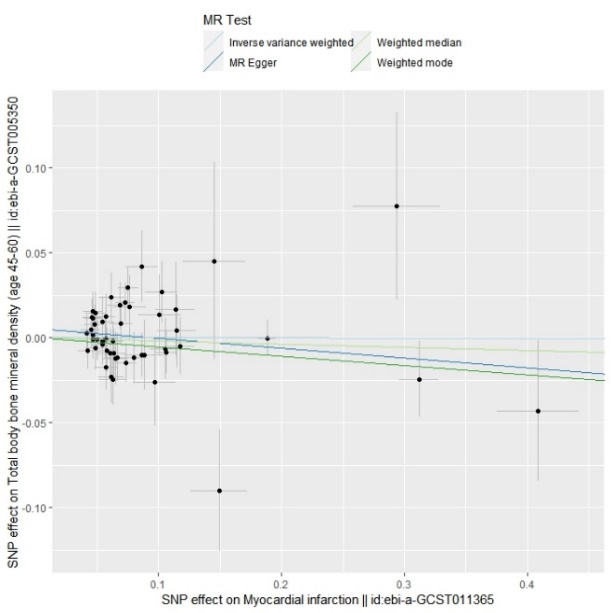


A B


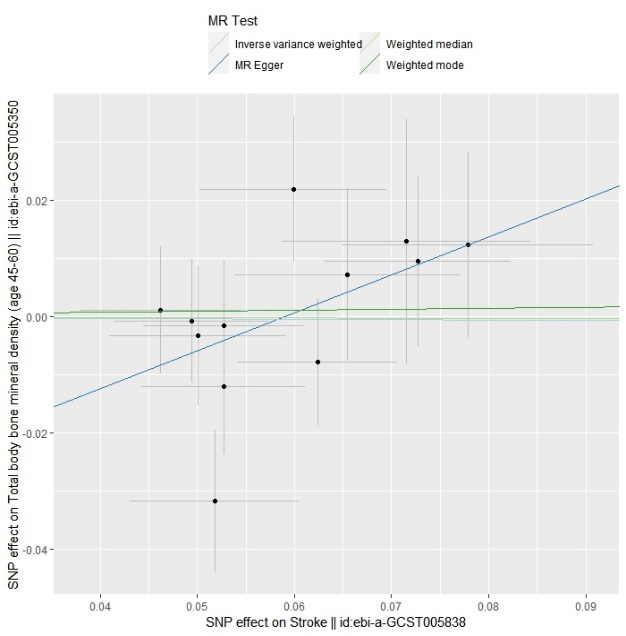


C

**Supplementary Figure S25.**

Scatter plots of causal estimates of exposure (Cardiovascular diseases) on Total body bone mineral density (age over 60). The slope of each line corresponding to the estimated MR effect in different models, including the conventional IVW, Weighted median, MR-Egger, Simple mode, and Weighted mode. (A): Coronary heart disease; (B): Myocardial infarction; (C): Stroke


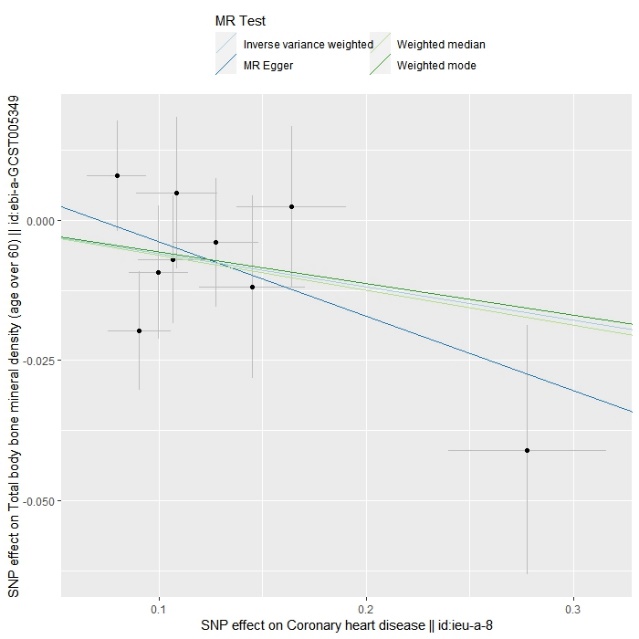

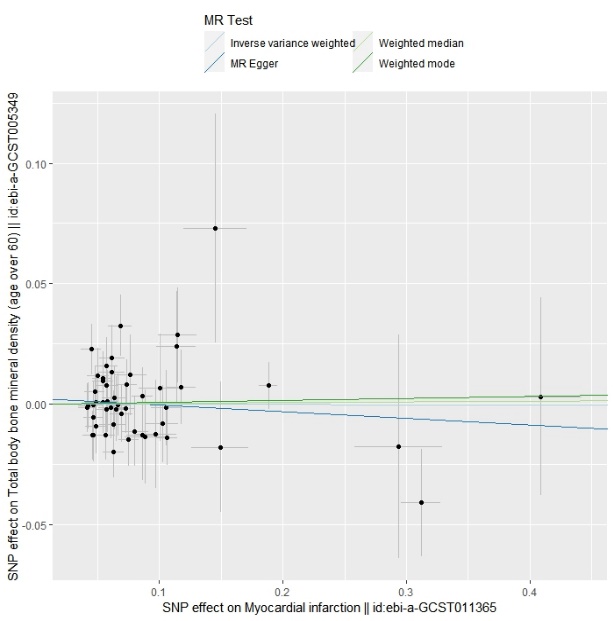


A B


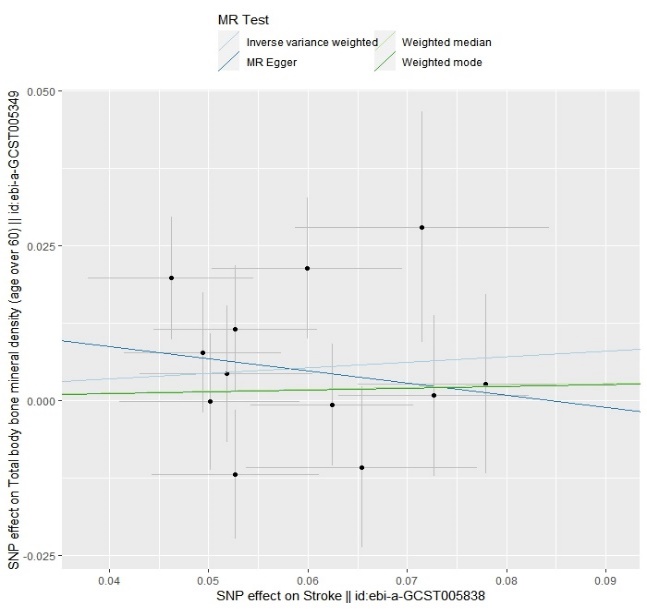


C

**Supplementary Figure S26.**

Leave-one-out stability tests causal estimates of exposure (Cardiovascular diseases) on Total body bone mineral density (age0-15). The slope of each line corresponding to the estimated MR effect in different models, including the conventional IVW, Weighted median, MR-Egger, Simple mode, and Weighted mode. (A): coronary heart disease; (B): myocardial infarction; (C): Stroke


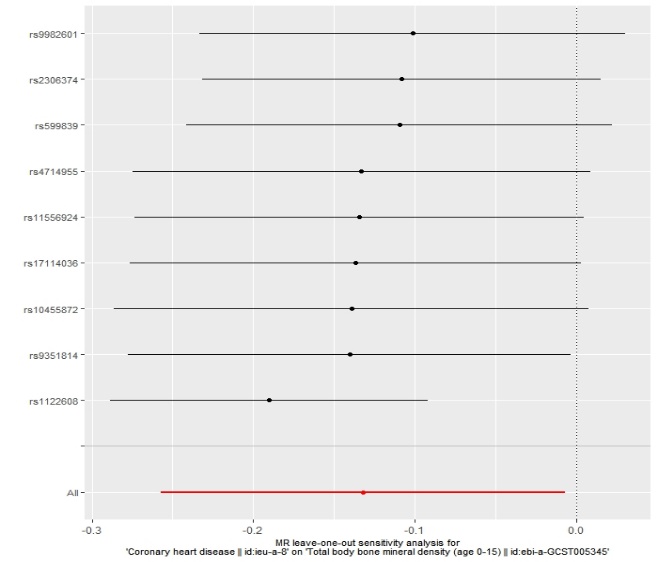

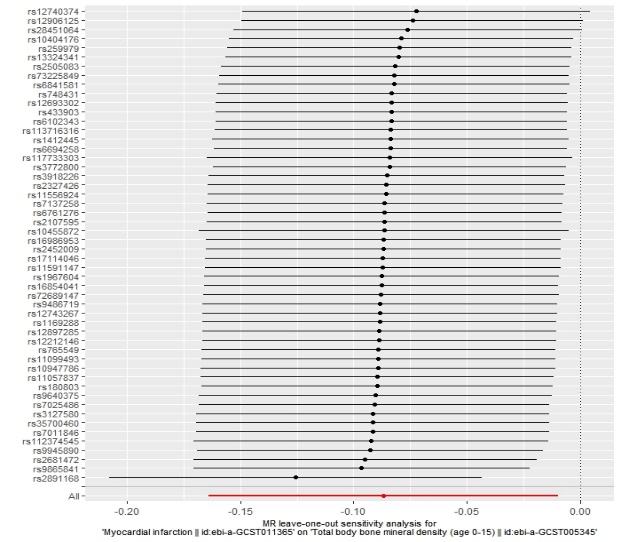


A B


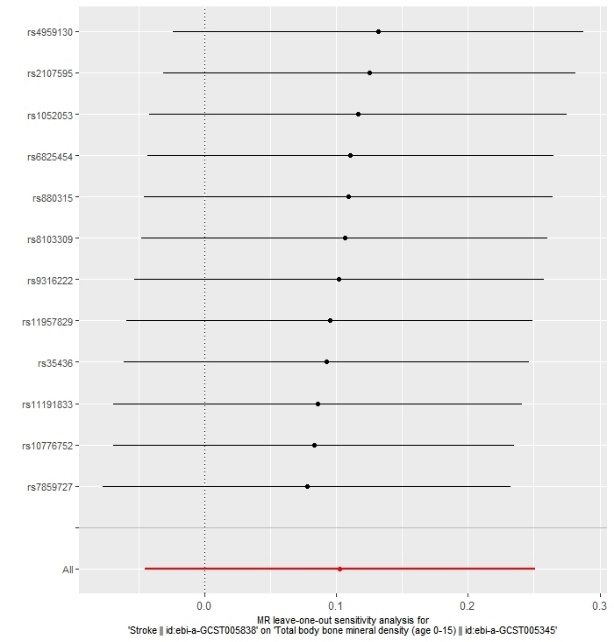


C

**Supplementary Figure S27.**

Leave-one-out stability tests causal estimates of exposure (Cardiovascular diseases) on Total body bone mineral density (age15-30). The slope of each line corresponding to the estimated MR effect in different models, including the conventional IVW, Weighted median, MR-Egger, Simple mode, and Weighted mode. (A): coronary heart disease; (B): myocardial infarction; (C): Stroke


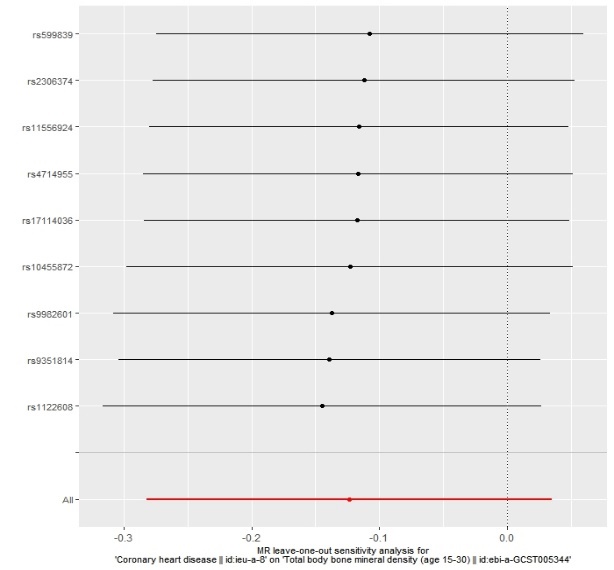

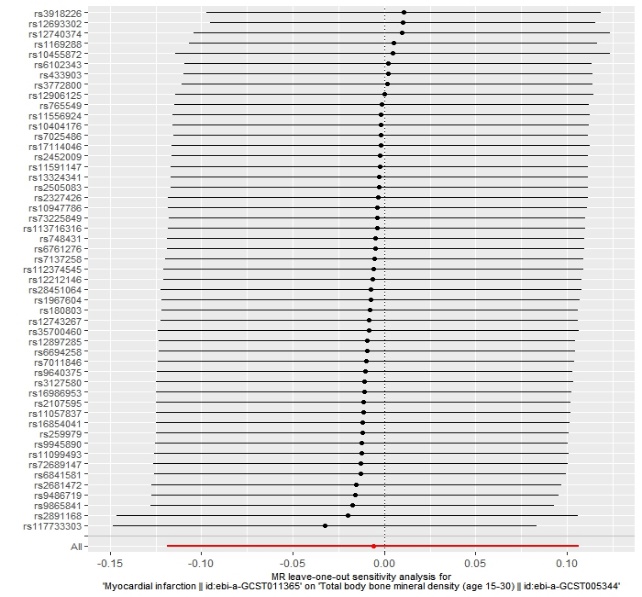


A B


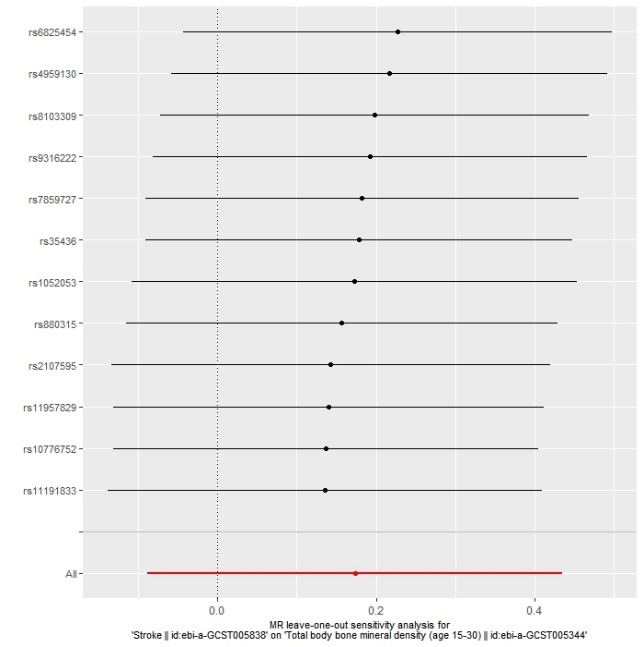


C

**Supplementary Figure S28.**

Leave-one-out stability tests causal estimates of exposure (Cardiovascular diseases) on Total body bone mineral density (age30-45). The slope of each line corresponding to the estimated MR effect in different models, including the conventional IVW, Weighted median, MR-Egger, Simple mode, and Weighted mode. (A): coronary heart disease; (B): myocardial infarction; (C): Stroke


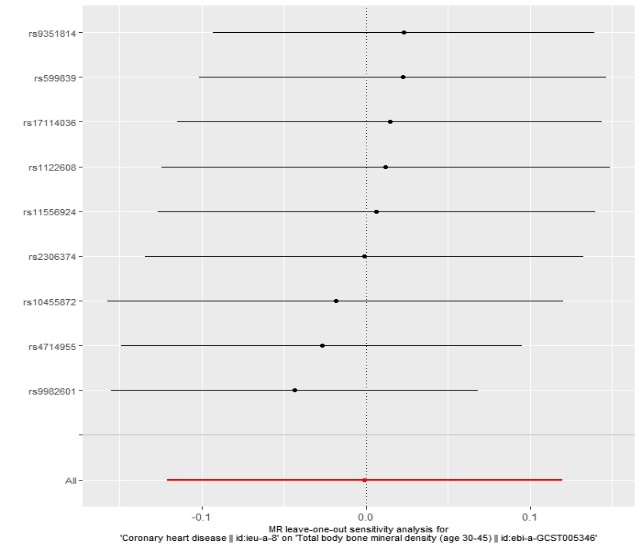

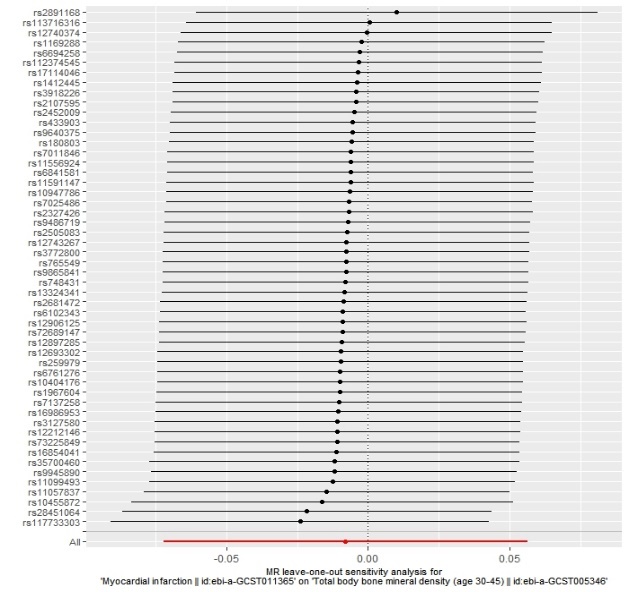


A B


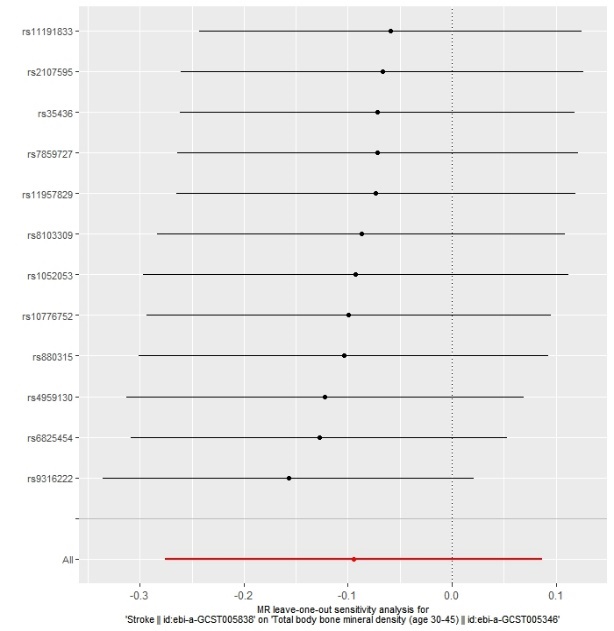


C

**Supplementary Figure S29.**

Leave-one-out stability tests causal estimates of exposure (Cardiovascular diseases) on Total body bone mineral density (age45-60). The slope of each line corresponding to the estimated MR effect in different models, including the conventional IVW, Weighted median, MR-Egger, Simple mode, and Weighted mode. (A): coronary heart disease; (B): myocardial infarction; (C): Stroke


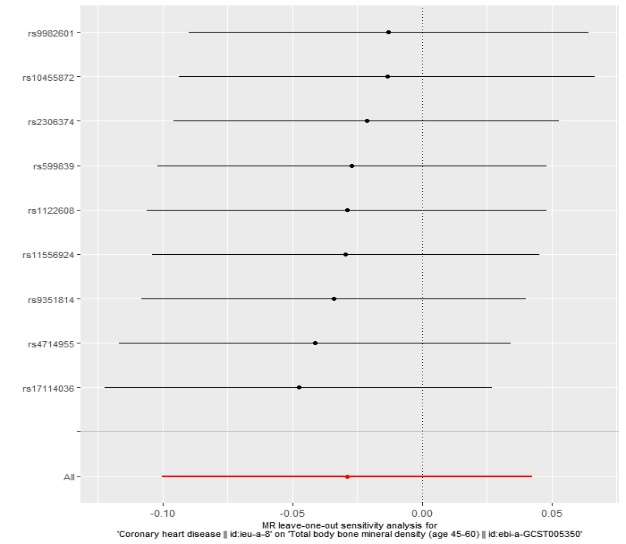

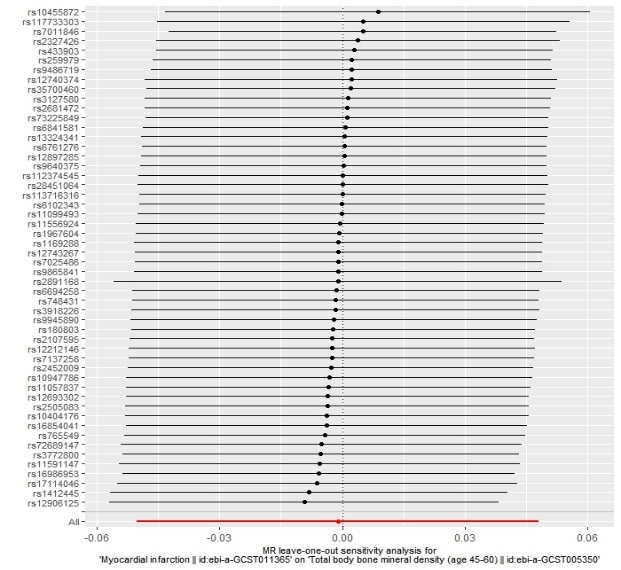


A B


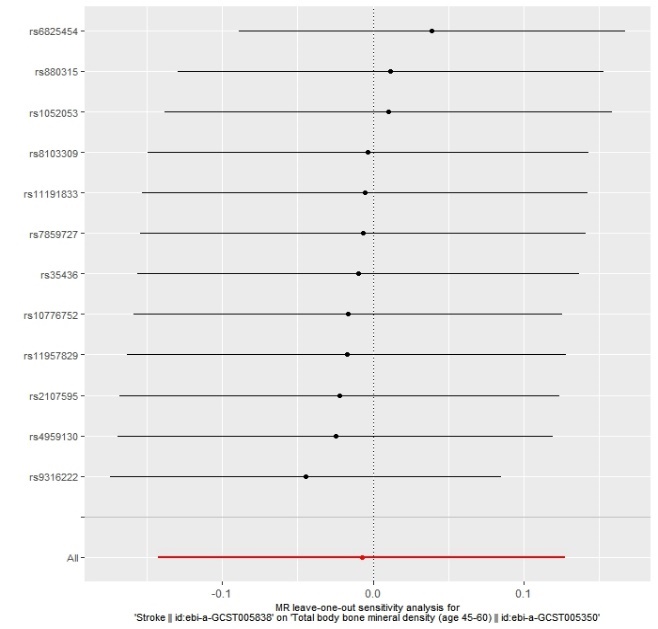


C

**Supplementary Figure S30.**

Leave-one-out stability tests causal estimates of exposure (Cardiovascular diseases) on Total body bone mineral density (age over 60). The slope of each line corresponding to the estimated MR effect in different models, including the conventional IVW, Weighted median, MR-Egger, Simple mode, and Weighted mode. (A): coronary heart disease; (B): myocardial infarction; (C): Stroke


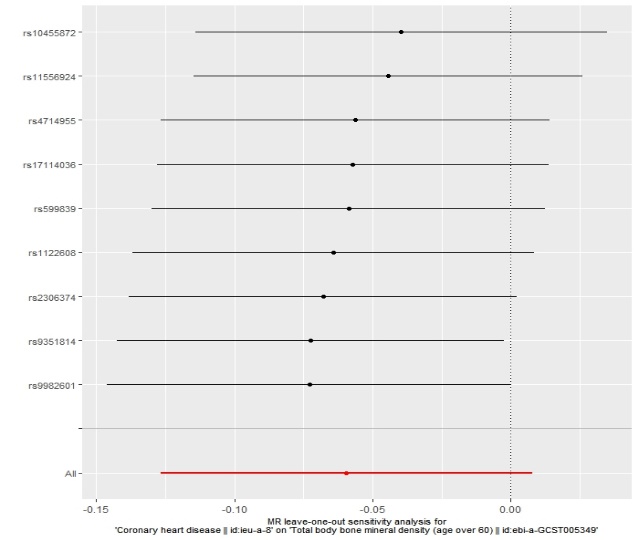

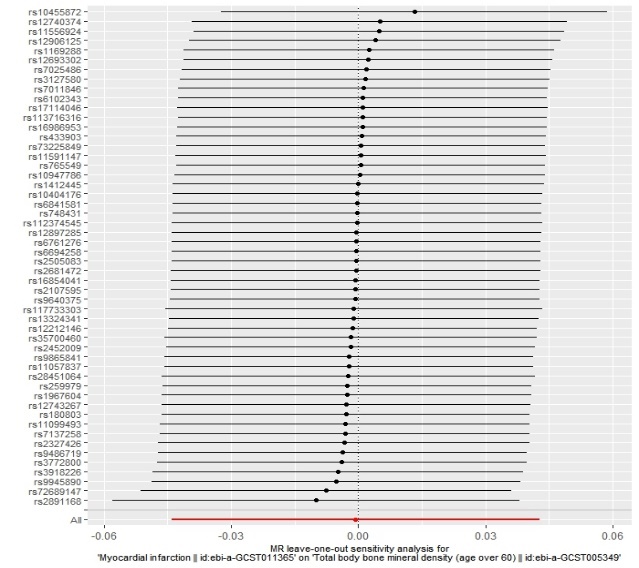


A B


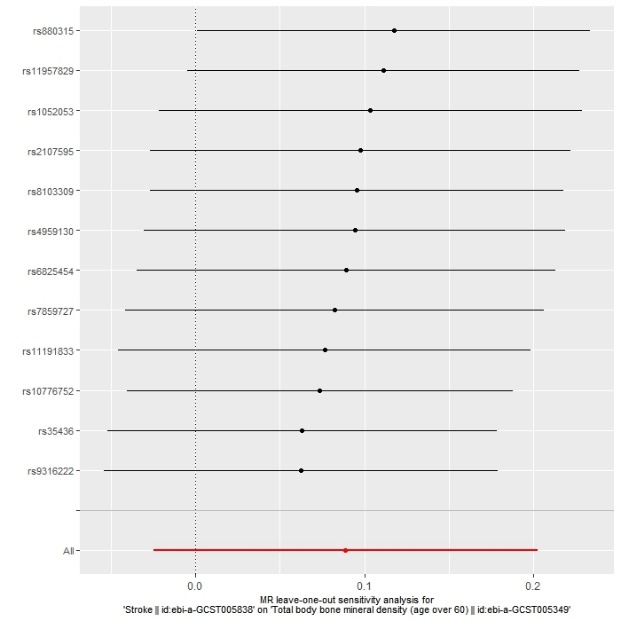


C

**Supplementary Figure S31.**

Forest plots of causal estimates of exposure (Cardiovascular diseases) on Total body bone mineral density (age0-15). The slope of each line corresponding to the estimated MR effect in different models, including the conventional IVW, Weighted median, MR-Egger, Simple mode, and Weighted mode. (A): coronary heart disease; (B): myocardial infarction; (C): Stroke


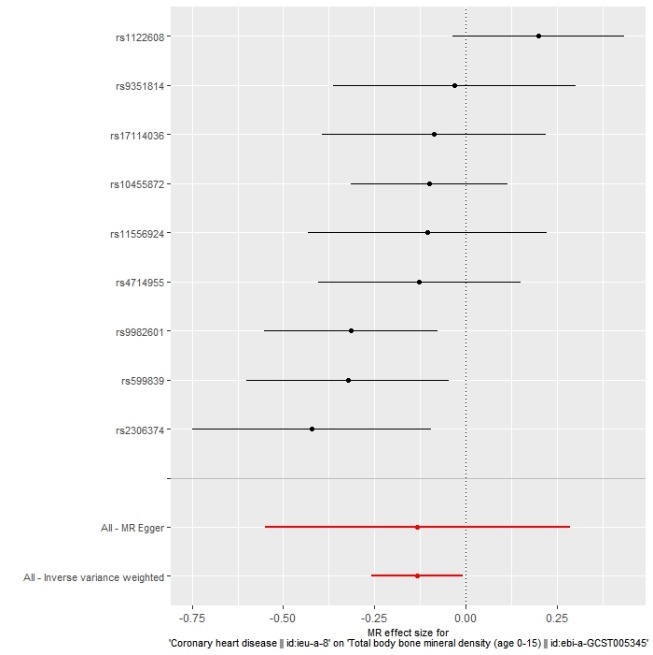

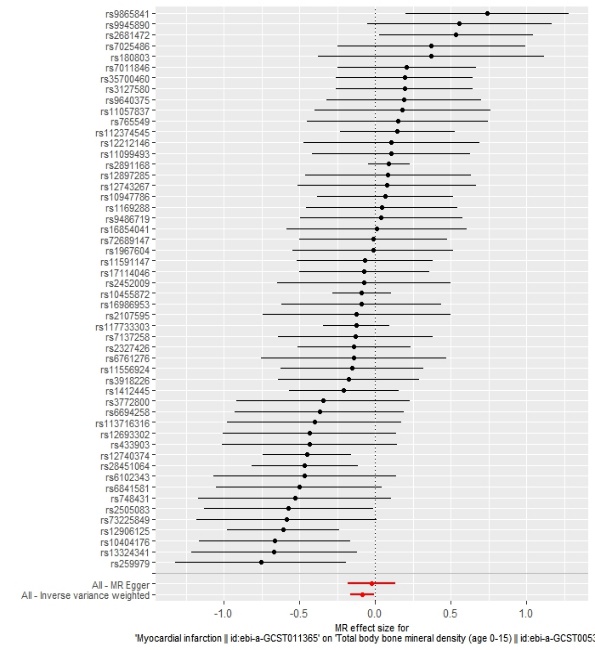


A B


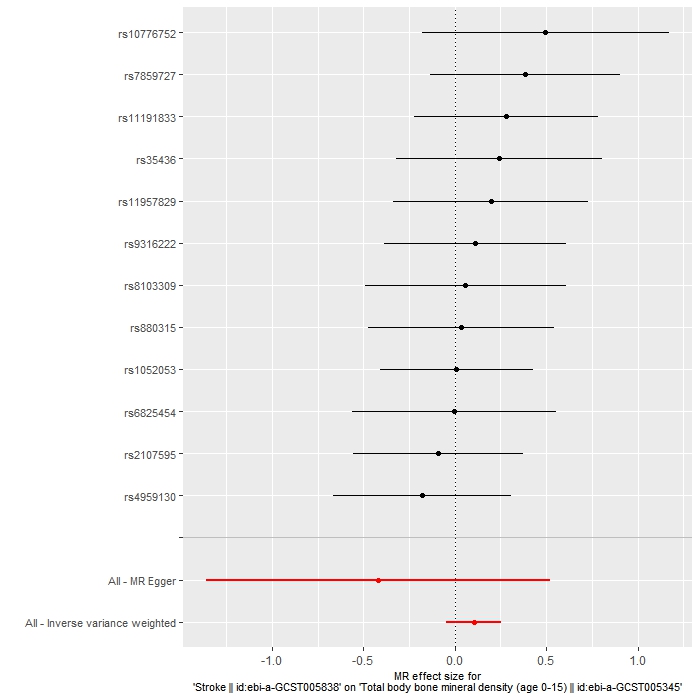


C

**Supplementary Figure S32.**

Forest plots of causal estimates of exposure (Cardiovascular diseases) on Total body bone mineral density (age15-30). The slope of each line corresponding to the estimated MR effect in different models, including the conventional IVW, Weighted median, MR-Egger, Simple mode, and Weighted mode. (A): coronary heart disease; (B): myocardial infarction; (C): Stroke


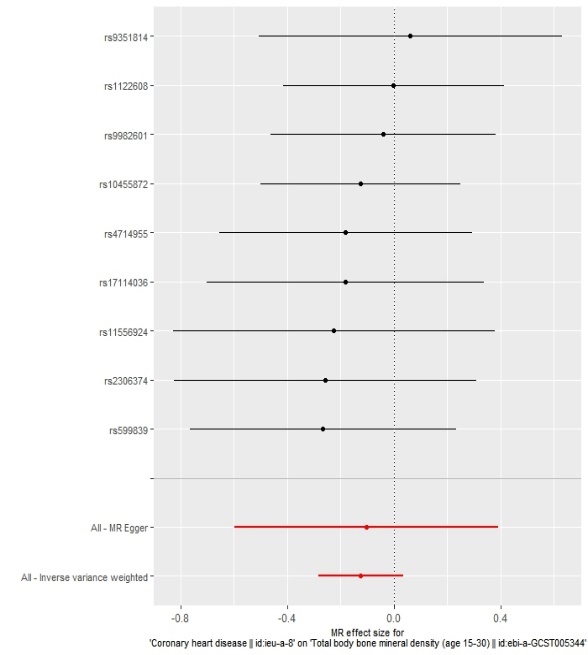

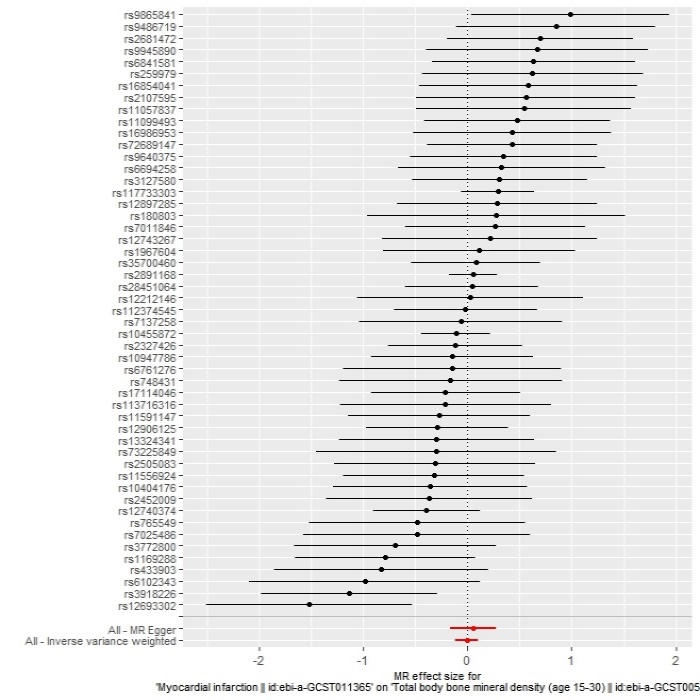


A B


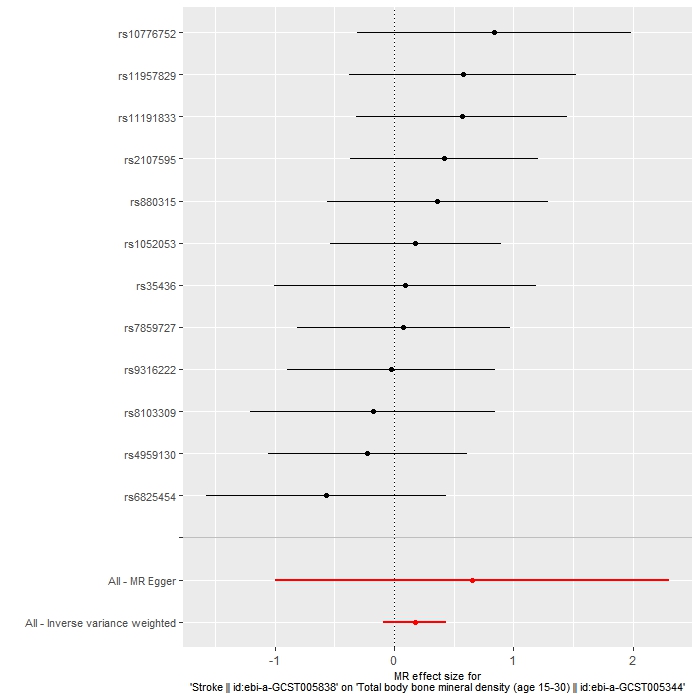


C

**Supplementary Figure S33.**

Forest plots of causal estimates of exposure (Cardiovascular diseases) on Total body bone mineral density (age30-45). The slope of each line corresponding to the estimated MR effect in different models, including the conventional IVW, Weighted median, MR-Egger, Simple mode, and Weighted mode. (A): coronary heart disease; (B): myocardial infarction; (C): Stroke


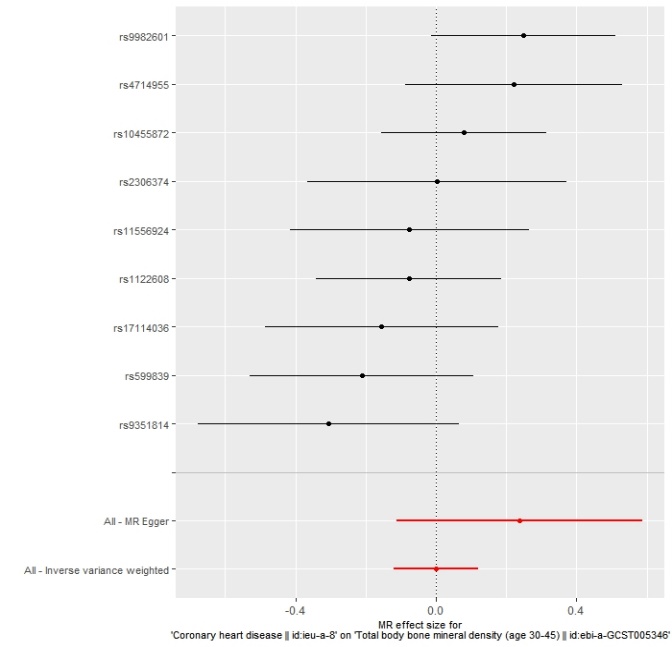

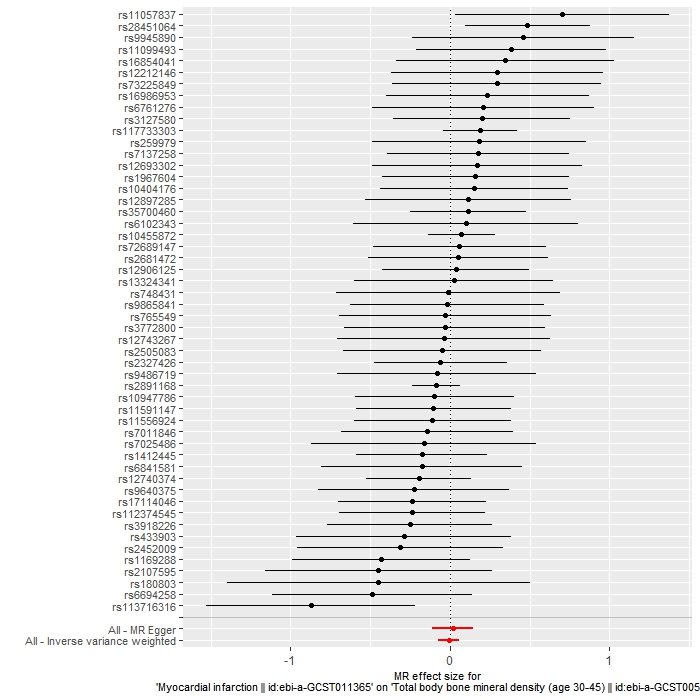


A B


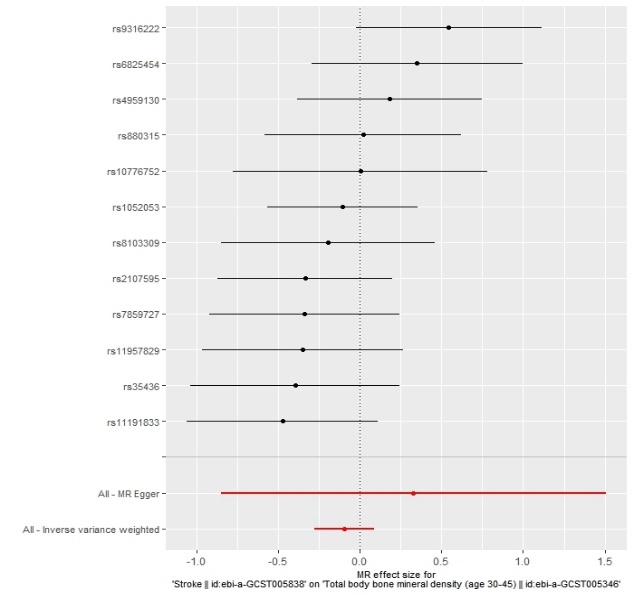


C

**Supplementary Figure S34.**

Forest plots of causal estimates of exposure (Cardiovascular diseases) on Total body bone mineral density (age45-60). The slope of each line corresponding to the estimated MR effect in different models, including the conventional IVW, Weighted median, MR-Egger, Simple mode, and Weighted mode. (A): coronary heart disease; (B): myocardial infarction; (C): Stroke


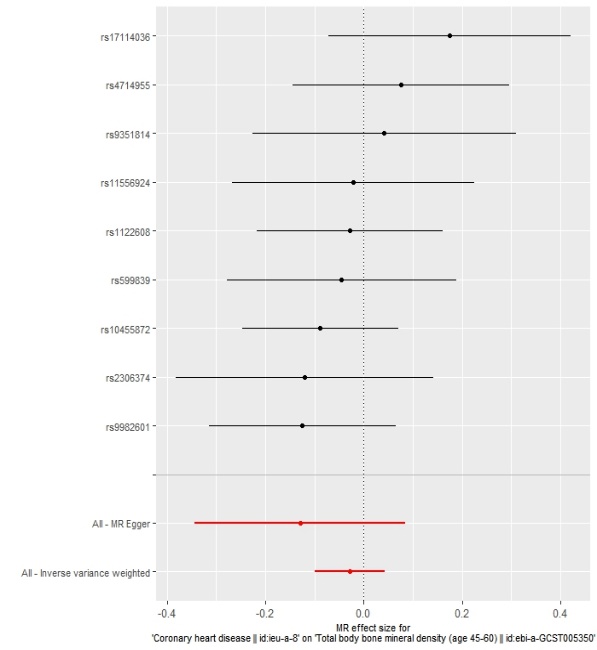


A B

C

**Supplementary Figure S35.**

Forest plots of causal estimates of exposure (Cardiovascular diseases) on Total body bone mineral density (age over 60). The slope of each line corresponding to the estimated MR effect in different models, including the conventional IVW, Weighted median, MR-Egger, Simple mode, and Weighted mode. (A): coronary heart disease; (B): myocardial infarction; (C): Stroke

A B

C

**Supplementary Figure S36.**

Funnel plots of causal estimates of exposure (Cardiovascular diseases) on Total body bone mineral density(age0-15). The slope of each line corresponding to the estimated MR effect in different models, including the conventional IVW, Weighted median, MR-Egger, Simple mode, and Weighted mode. (A): coronary heart disease; (B): myocardial infarction; (C): Stroke

MR effect size of for MR effect size of for

“Coronary heart disease” on “Total body bone mineral density(age0-15)” “Myocardial infarction” on “Total body bone mineral density(age0-15)”

A B

MR effect size of for

“Stroke” on “Total body bone mineral density(age0-15)”

C

**Supplementary Figure S37.**

Funnel plots of causal estimates of exposure (Cardiovascular diseases) on Total body bone mineral density(age15-30). The slope of each line corresponding to the estimated MR effect in different models, including the conventional IVW, Weighted median, MR-Egger, Simple mode, and Weighted mode. (A): coronary heart disease; (B): myocardial infarction; (C): Stroke

MR effect size of for MR effect size of for

“Coronary heart disease” on “Total body bone mineral density(age15-30)” “Myocardial infarction” on “Total body bone mineral density(age15-30)”

A B

MR effect size of for

“Stroke” on “Total body bone mineral density(age15-30)”

C

**Supplementary Figure S38.**

Funnel plots of causal estimates of exposure (Cardiovascular diseases) on Total body bone mineral density(age30-45). The slope of each line corresponding to the estimated MR effect in different models, including the conventional IVW, Weighted median, MR-Egger, Simple mode, and Weighted mode. (A): coronary heart disease; (B): myocardial infarction; (C): Stroke

MR effect size of for MR effect size of for

“Coronary heart disease” on “Total body bone mineral density(age30-45)” “Myocardial infarction” on “Total body bone mineral density(age30-45)”

A B

MR effect size of for

“Stroke” on “Total body bone mineral density(age30-45)”

C

**Supplementary Figure S39.**

Funnel plots of causal estimates of exposure (Cardiovascular diseases) on Total body bone mineral density(age45-60). The slope of each line corresponding to the estimated MR effect in different models, including the conventional IVW, Weighted median, MR-Egger, Simple mode, and Weighted mode. (A): coronary heart disease; (B): myocardial infarction; (C): Stroke

MR effect size of for MR effect size of for

“Coronary heart disease” on “Total body bone mineral density(age45-60)” “Myocardial infarction” on “Total body bone mineral density(age45-60)”

A B

MR effect size of for

“Stroke” on “Total body bone mineral density(age45-60)”

C

**Supplementary Figure S40.**

Funnel plots of causal estimates of exposure (Cardiovascular diseases) on Total body bone mineral density(age over 60). The slope of each line corresponding to the estimated MR effect in different models, including the conventional IVW, Weighted median, MR-Egger, Simple mode, and Weighted mode. (A): coronary heart disease; (B): myocardial infarction; (C): Stroke

MR effect size of for MR effect size of for

“Coronary heart disease” on “Total body bone mineral density(age over 60)” “Myocardial infarction” on “Total body bone mineral density(age over 60)”

A B

MR effect size of for

“Stroke” on “Total body bone mineral density(age over 60)”

C

**Supplementary Figure S41.**

Scatter plots of causal estimates of exposure (Bone density) on coronary heart disease. The slope of each line corresponding to the estimated MR effect in different models, including the conventional IVW, Weighted median, MR-Egger, Simple mode, and Weighted mode. (A): Forearm bone mineral density(p＜5×10^−8^); (B): Forearm bone mineral density(p＜5×10^−6^); (C): Femoral neck mineral density; (D): Heel bone mineral density;(E): Lumbar spine bone density;(F): Total body bone mineral density

A B

C D

E F

**Supplementary Figure S42.**

Scatter plots of causal estimates of exposure (Bone density) on myocardial infarction. The slope of each line corresponding to the estimated MR effect in different models, including the conventional IVW, Weighted median, MR-Egger, Simple mode, and Weighted mode. (A): Forearm bone mineral density(p＜5×10^−8^); (B): Forearm bone mineral density(p＜5×10^−6^); (C): Femoral neck mineral density; (D): Heel bone mineral density;(E): Lumbar spine bone density;(F): Total body bone mineral density.

A B

C D

E F

**Supplementary Figure S43.**

Scatter plots of causal estimates of exposure (Bone density) on stroke. The slope of each line corresponding to the estimated MR effect in different models, including the conventional IVW, Weighted median, MR-Egger, Simple mode, and Weighted mode. (A): Forearm bone mineral density(p＜5×10^−8^); (B): Forearm bone mineral density(p＜5×10^−6^); (C): Femoral neck mineral density; (D): Heel bone mineral density;(E): Lumbar spine bone density;(F): Total body bone mineral density.

A B

C D

E F

**Supplementary Figure S44.**

Leave-one-out stability tests causal estimates of exposure (Bone density) on coronary heart disease. The slope of each line corresponding to the estimated MR effect in different models, including the conventional IVW, Weighted median, MR-Egger, Simple mode, and Weighted mode. (A): Forearm bone mineral density(p＜5×10^−8^); (B): Forearm bone mineral density(p＜5×10^−6^); (C): Femoral neck mineral density; (D): Heel bone mineral density;(E): Lumbar spine bone density;(F): Total body bone mineral density.

A B

C D

E F

**Supplementary Figure S45.**

Leave-one-out stability tests causal estimates of exposure (Bone density) on myocardial infarction. The slope of each line corresponding to the estimated MR effect in different models, including the conventional IVW, Weighted median, MR-Egger, Simple mode, and Weighted mode. (A): Forearm bone mineral density(p＜5×10^−8^); (B): Forearm bone mineral density(p＜5×10^−6^); (C): Femoral neck mineral density; (D): Heel bone mineral density;(E): Lumbar spine bone density;(F): Total body bone mineral density.

A B

C D

E F

**Supplementary Figure S46.**

Leave-one-out stability tests causal estimates of exposure (Bone density) on stroke. The slope of each line corresponding to the estimated MR effect in different models, including the conventional IVW, Weighted median, MR-Egger, Simple mode, and Weighted mode. (A): Forearm bone mineral density(p＜5×10^−8^); (B): Forearm bone mineral density(p＜5×10^−6^); (C): Femoral neck mineral density; (D): Heel bone mineral density;(E): Lumbar spine bone density;(F): Total body bone mineral density.

A B

C D

E F

**Supplementary Figure S47.**

Forest plots of causal estimates of exposure (Bone density) on coronary heart disease. The slope of each line corresponding to the estimated MR effect in different models, including the conventional IVW, Weighted median, MR-Egger, Simple mode, and Weighted mode. (A): Forearm bone mineral density(p＜5×10^−8^); (B): Forearm bone mineral density(p＜5×10^−6^); (C): Femoral neck mineral density; (D): Heel bone mineral density;(E): Lumbar spine bone density;(F): Total body bone mineral density.

A B

C D

E F

**Supplementary Figure S48.**

Forest plots of causal estimates of exposure (Bone density) on myocardial infarction. The slope of each line corresponding to the estimated MR effect in different models, including the conventional IVW, Weighted median, MR-Egger, Simple mode, and Weighted mode. (A): Forearm bone mineral density(p＜5×10^−8^); (B): Forearm bone mineral density(p＜5×10^−6^); (C): Femoral neck mineral density; (D): Heel bone mineral density;(E): Lumbar spine bone density;(F): Total body bone mineral density.

A B

C D

E F

**Supplementary Figure S49.**

Forest plots of causal estimates of exposure (Bone density) on stroke. The slope of each line corresponding to the estimated MR effect in different models, including the conventional IVW, Weighted median, MR-Egger, Simple mode, and Weighted mode. (A): Forearm bone mineral density(p＜5×10^−8^); (B): Forearm bone mineral density(p＜5×10^−6^); (C): Femoral neck mineral density; (D): Heel bone mineral density;(E): Lumbar spine bone density;(F): Total body bone mineral density.

A B

C D

E F

**Supplementary Figure S50.**

Funnel plots of causal estimates of exposure (Bone density) on coronary heart disease. The slope of each line corresponding to the estimated MR effect in different models, including the conventional IVW, Weighted median, MR-Egger, Simple mode, and Weighted mode. (A): Forearm bone mineral density(p＜5×10^−8^); (B): Forearm bone mineral density(p＜5×10^−6^); (C): Femoral neck mineral density; (D): Heel bone mineral density;(E): Lumbar spine bone density;(F): Total body bone mineral density.

MR effect size of for MR effect size of for

“Forearm bone mineral density(p＜5×10^−8^)” on “coronary heart disease” “Forearm bone mineral density(p＜5×10^−6^)” on “coronary heart disease”

A B

MR effect size of for MR effect size of for

“Femoral neck mineral density” on “coronary heart disease” “Heel bone mineral density” on “coronary heart disease”

C D

MR effect size of for MR effect size of for

“Lumbar spine bone density” on “coronary heart disease” “Total body bone mineral density” on “coronary heart disease”

E F

**Supplementary Figure S51.**

Funnel plots of causal estimates of exposure (Bone density) on myocardial infarction. The slope of each line corresponding to the estimated MR effect in different models, including the conventional IVW, Weighted median, MR-Egger, Simple mode, and Weighted mode. (A): Forearm bone mineral density(p＜5×10^−8^); (B): Forearm bone mineral density(p＜5×10^−6^); (C): Femoral neck mineral density; (D): Heel bone mineral density;(E): Lumbar spine bone density;(F): Total body bone mineral density.

MR effect size of for MR effect size of for

“Forearm bone mineral density(p＜5×10^−8^)” on “myocardial infarction” “Forearm bone mineral density(p＜5×10^−6^)” on “myocardial infarction”

A B

MR effect size of for MR effect size of for

“Femoral neck mineral density” on “myocardial infarction” “Heel bone mineral density” on “myocardial infarction”

C D

MR effect size of for MR effect size of for

“Lumbar spine bone density” on “myocardial infarction” “Total body bone mineral density” on “myocardial infarction”

E F

**Supplementary Figure S52.**

Funnel plots of causal estimates of exposure (Bone density) on stroke. The slope of each line corresponding to the estimated MR effect in different models, including the conventional IVW, Weighted median, MR-Egger, Simple mode, and Weighted mode. (A): Forearm bone mineral density(p＜5×10^−8^); (B): Forearm bone mineral density(p＜5×10^−6^); (C): Femoral neck mineral density; (D): Heel bone mineral density;(E): Lumbar spine bone density;(F): Total body bone mineral density.

MR effect size of for MR effect size of for

“Forearm bone mineral density(p＜5×10^−8^)” on “stroke” “Forearm bone mineral density(p＜5×10^−6^)” on stroke”

A B

MR effect size of for MR effect size of for

“Femoral neck mineral density” on “stroke” “Heel bone mineral density” on “stroke”

C D

MR effect size of for MR effect size of for

“Lumbar spine bone density” on stroke” “Total body bone mineral density” on stroke”

E F
